# The COVID-19 resilience of a continental welfare regime - nowcasting the distributional impact of the crisis

**DOI:** 10.1007/s10888-021-09524-4

**Published:** 2022-02-22

**Authors:** Denisa M. Sologon, Cathal O’Donoghue, Iryna Kyzyma, Jinjing Li, Jules Linden, Raymond Wagener

**Affiliations:** 1 Luxembourg Institute of Socio-Economic Research (LISER), Esch-sur-Alzette, Luxembourg; 2 National University of Ireland, Galway (NUIG), Galway, Ireland; 3 University of Canberra, Canberra, Australia; 4 IGSS, Luxembourg, Luxembourg

**Keywords:** COVID-19, Nowcasting, Microsimulation, Income inequality, Tax-benefit policy

## Abstract

We evaluate the COVID-19 resilience of a Continental welfare regime by nowcasting the implications of the shock and its associated policy responses on the distribution of household incomes over the whole of 2020. Our approach relies on a dynamic microsimulation modelling that combines a household income generation model estimated on the latest EU-SILC wave with novel nowcasting techniques to calibrate the simulations using external macro controls which reflect the macroeconomic climate during the crisis. We focus on Luxembourg, a country that introduced minor tweaks to the existing tax-benefit system, which has a strong social insurance focus that gave certainty during the crisis. We find the system was well-equipped ahead of the crisis to cushion household incomes against job losses. The income-support policy changes were effective in cushioning household incomes and mitigating an increase in income inequality, allowing average household disposable income and inequality levels to bounce back to pre-crisis levels in the last quarter of 2020. The share of labour incomes dropped, but was compensated by an increase in benefits, reflecting the cushioning effect of the transfer system. Overall market incomes dropped and became more unequal. Their disequalizing evolution was matched by an increase in redistribution, driven by an increase in the generosity of benefits and larger access to benefits. The nowcasting model is a “near” real-time analysis and decision support tool to monitor the recovery, scalable to other countries with high applicability for policymakers.

**Supplementary Information:**

The online version contains supplementary material available at doi:10.1007/s10888-021-09524-4.

## 1 Introduction

The novel coronavirus, which reached Europe in February 2020, forced governments of many countries to undertake unprecedented measures to contain the pandemic, including full or partial lockdowns, involving travel restrictions, school and workplace closings, bans on public events, restrictions on gatherings, and 'stay at home' requirements etc. (Hale et al. 2020). These containment measures resulted in a wide range of adverse economic consequences in many countries, leading to a persistent recession (Eichenbaum et al. 2020), which by its scope is expected to outweigh the Great Recession of 2008. An immediate question that arises is how the macroeconomic shock related to COVID-19, combined with the mitigating policy response, has affected the distribution of household equivalized disposable incomes.

Given the significant asymmetric nature of the shock, there has been a substantial analytical response to it (O'Donoghue et al. 2020; Brewer and Gardiner 2020; Li et al. 2020; Bruckmeier et al. 2021; Almeida et al. 2021). Due to the nature and scale of the shock many countries had to implement substantial policy responses to enhance the mitigating impact of policy (e.g. Ireland, UK, Italy, Australia). In this paper, we consider the situation of Luxembourg, a country whose social protection system was relatively resilient and where minor policy responses were made. We aim to evaluate the impact of the COVID-19 crisis and the associated policy responses on the distribution of household incomes in Luxembourg through the whole of 2020, namely at the peak of the crisis in April, as well as in the second, third and fourth quarter of 2020.

Luxembourg is a country with well-established fiscal and social policy instruments and regulators, which were efficient in mitigating the impacts of previous crises on the economy in general and on welfare of individuals in particular (Dolls et al. 2012; Dolls et al. 2020; Paulus and Tasseva 2020). In the COVID crisis, while some countries had to introduce radically different policies from their existing systems (e.g. Ireland, the UK, Italy, Australia), Luxembourg made relatively minor changes in exiting policy instruments, with changes focused primarily on the administration of the system rather than changes in benefits rules. The presence of these instruments and the availability of financial resources formed strong economic and legislative prerequisites for a fast policy response during the COVID-19 pandemic, whose distributional outcomes we evaluate in this paper.[1]

[1] As shown by Dolls et al. (2020), automatic stabilizers are much stronger in Nordic and Continental Europe (which also have a higher GDP per capita) than in Eastern or Southern European countries. Almeida et al. (2021) further reveal that richer European countries were more successful in reducing COVID-related losses in household disposable incomes and mitigating an increase in income poverty than poorer countries.

The main challenge in assessing the immediate impact of the COVID-19 pandemic on individuals' incomes and welfare lies in the availability of data. Large-scale representative surveys on income and living conditions, which are typically used for the distributional analysis, become available with a one- or two-year delay. When a sudden economic shock hits, these data become obsolete. Governments, however, need the knowledge of the real-time income situation of individuals at the moment of the crisis (Jenkins et al. 2012; Salgado et al. 2014; Chetty et al. 2020). We overcome this challenge by applying a nowcasting approach based on dynamic microsimulation using up-to-date official statistics on the shifts in sectoral employment and tax-benefit policies. These are used to calibrate the simulations from a household income generation model (see Sologon et al. 2021) estimated on the latest available survey data from the European Union Statistics on Income and Living Conditions (EU-SILC). This approach allows for greater heterogeneity in responses compared to the simpler transition matrix method which relies on employment transitions (O'Donoghue and Sologon 2021). Following the calibration approach of Li and O'Donoghue (2014), we align household incomes with the current macroeconomic situation, similarly to O'Donoghue et al. (2020). We then use this data to analyse the "near" real-time effects of the COVID-19 crisis on individual incomes and welfare at the peak of the crisis outbreak in April 2020 and in later quarters of 2020.

Our paper contributes to an increasing literature on the economic consequences of the COVID-19 pandemic for the welfare of individuals and the households they live in. Among the studies on the distributional effects of the crisis we mention Brewer and Gardiner (2020), Brewer and Tasseva (2021), and Bronka et al. (2020) in the UK, Beirne et al. (2020) and O'Donoghue et al. (2020) in Ireland, Figari and Fiorio (2020) in Italy, Bruckmeier et al. (2021) in Germany, Li et al. (2020) in Australia, and Almeida et al. (2021) for 27 EU countries. In Italy, despite the presence of automatic stabilizers and the introduction of new policy instruments, researchers document an increase in inequality in household disposable incomes during the first months of the COVID-19 outbreak. In contrast, in Australia, Ireland, and Germany the introduction of income support programmes in response to the crisis prevented a drop in household disposable incomes at the bottom of the distribution, which, in combination with the suppressed incomes at the top of the distribution, led to a decline in inequality (and poverty). The evidence for the UK is quite diverse, with Brewer and Tasseva (2021) reporting an expected increase (using a fixed poverty line) and Bronka et al. (2020) a decrease in poverty as a consequence of the COVID-19 crisis.[2]

Our findings show that, following the COVID-19 outbreak, market incomes dropped along the distribution, with a decline being the largest in April (when Luxembourg entered a strict lockdown), somewhat smaller in May and June (following the graduate lifting of the lockdown measures), and gradually disappearing towards the end of 2020. Market incomes also became more unequally distributed, with the losses being larger in the middle of the

[2]While comparing the findings from these studies one should keep in mind that they rely on different modelling techniques, different data sources for calibration, and that the analyses are conducted at different stages of the crisis and have different accounting periods. For example, in the case of the UK, Brewer and Tasseva (2021) estimate multinominal logit models using actual data from the Understanding Society COVID-19 Study collected in April/May 2020 to simulate employment and earnings shocks in the Family Resources Survey 2018/2019. To predict the effects of the COVID-19 on the economy, Bronka et al. (2020) use the scenario parameters for the supply and demand shocks derived from the ad-hoc survey of economists with UK affiliations, which they incorporate in an input-output model. One important difference is that the effect of the crisis is analysed at different stages and is measured over different periods. Whereas Brewer and Tasseva (2021) assesses the impact of the crisis in certain periods of 2020 (e.g. April and May 2020), Bronka et al. (2020) estimate the effect in the full-financial year 2020/2021.

distribution than in its upper or lower tails. The decline in market incomes, however, was largely cushioned by an increase in redistribution through the tax-benefit system.

We find that the Luxembourg tax-benefit system was well-equipped ahead of the crisis, which allows it to cushion losses in market incomes and mitigate an increase in inequality of household disposable incomes even in the absence of COVID-specific policy measures. This fact distinguishes Luxembourg from other developed countries, where softening of the distributional consequences of the COVID-19 crisis became possible mainly thanks to the introduction of discretionary policy measures (e.g. Ireland, the UK).

Luxembourg is one of the only countries analysed during the COVID-19 crisis which showed that the existing system was resilient. One of the main contributions of our study is to showcase the structure of the system using an appropriate methodology, in the first study of its kind in Luxembourg. The main lesson is that it is not necessarily about the financing of income losses when the crisis hits, because other countries put in generous systems when they needed them; it is about building resilient systems. Luxembourg had this system by default. This is the direction many countries (e.g. Ireland) are taking now to make the systems more resilient in the future, to return to generous time-limited earnings-related unemployment benefits.

The methodological advantage of our study is that we combine dynamic microsimulation and nowcasting techniques to obtain “near” real-time household income data, which we then use to evaluate the distributional consequences of the COVID-19 crisis throughout 2020. The model provides a “near” real-time analysis and serves as a decision support tool to monitor the recovery, with high applicability for policymakers. In addition, the infrastructure is scalable to other countries as it relies on a flexible income generation model, on comparative cross-national survey data and macro alignment statistics, and the pan-European tax-benefit model, EUROMOD, enhancing its applicability and policy relevance.

## 2 Macroeconomic background

The first person infected with COVID-19 was registered in Luxembourg on February 29, 2020.[3] In response to increasing numbers of infections, the government introduced a set of lockdown measures from March 16, closing all educational facilities and non-essential businesses, encouraging remote work whenever possible, restricting gatherings, and limiting national and international travel.[4] Due to those measures, Luxembourg managed to avoid a severe outbreak of COVID-19 in spring and the measures started being lifted from April 20, with the construction sector and all related businesses coming back to work first. The gradual exit plan lasted until mid-July, when most of the restrictions were abolished except of the ban for large public gatherings and maintenance of sanitary measures. In November, the number of COVID-19 cases rose again and the government introduced a curfew and a partial lockdown affecting leisure activities, such as cinemas, sports facilities, restaurants and bars. On December 26, all non-essential businesses were closed.

Following the lockdown measures introduced in Luxembourg and the developments of the COVID-19 pandemic around the world, Luxembourg’s economy shrank by 7.8 percent

[3]See RTL Today - Man quarantined in CHL: First case of coronavirus confirmed in Luxembourg, Charleroi Airport increasing precautionary measures. Extracted from https://today.rtl.lu/news/luxembourg/a/1476925.html on December 3, 2020.

[4]See RTL Today - All bars and restaurants to close, 81 confirmed cases. Extracted from https://today.rtl.lu/news/luxembourg/a/1483941.html on December 3, 2020.

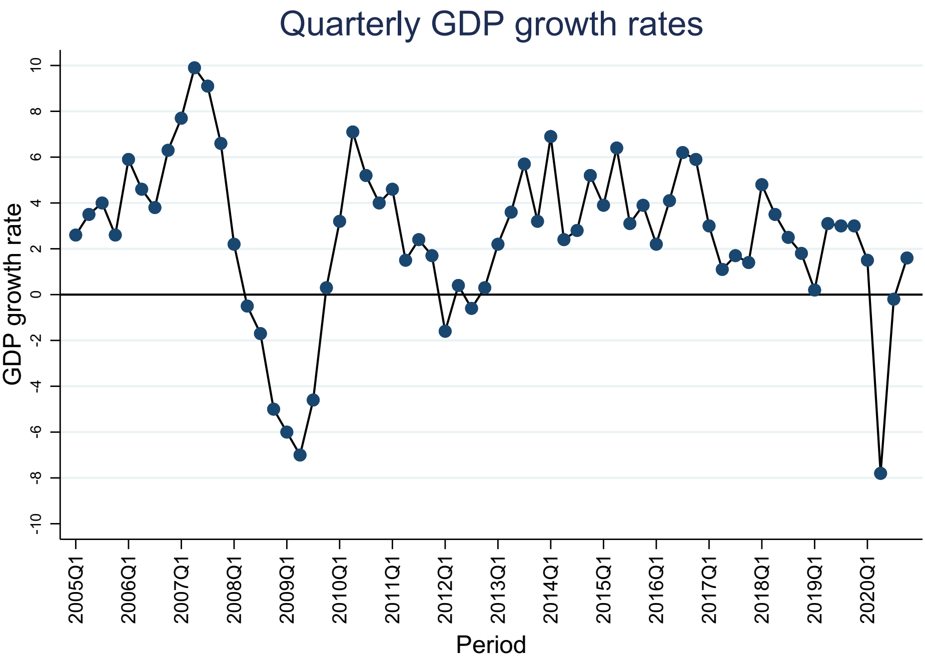

**Fig. 1** Percentage growth of GDP in market prices (one quarter compared to the same quarter in the previous year). Note: Extracted from https://statistiques.public.lu/en/index.html on July 21, 2021

in the second quarter of 2020 as compared to the analogous period in the previous year (Fig. 1). This was the largest decline in the country's quarterly GDP growth rate over recent decades, exceeding the size of the GDP decline during the Great Recession of 2008/2009. The decline, however, was much smaller in the third quarter of 2020 being followed by modest economic growth in Q4.

Similarly to other countries, a drastic decline in GDP growth rates was accompanied by an increase in unemployment (Fig. 2). The unemployment rate rose in Luxembourg between February and March, hit its highest level in April, and started slowly decreasing afterwards. By the end of December, however, it was still approximately 1 percentage point higher as compared to the pre-crisis level.

In addition to the increase in the percentage of workers who formally lost their jobs, a substantial portion of employees were forced into short-time work (STW).[5] Under short-time work, the government takes over 80 percent of the remuneration costs for hours not worked by employees who had their working hours involuntarily reduced, under the condition that they remain formally employed with their current employer and their working hours increase as soon as the economic situation improves. Figure 2 shows an adjusted unemployment rate which accounts for the share of employees on short-time work, providing a more nuanced view of the impact of COVID-19 on employment. The share of employees affected by COVID-19 far exceeded that of those affected during the Great Recession. Almost 34 percent of employed individuals residing in Luxembourg

[5] A description of short-time work and other relevant policies is provided in Table A-2 in the Supplementary Material

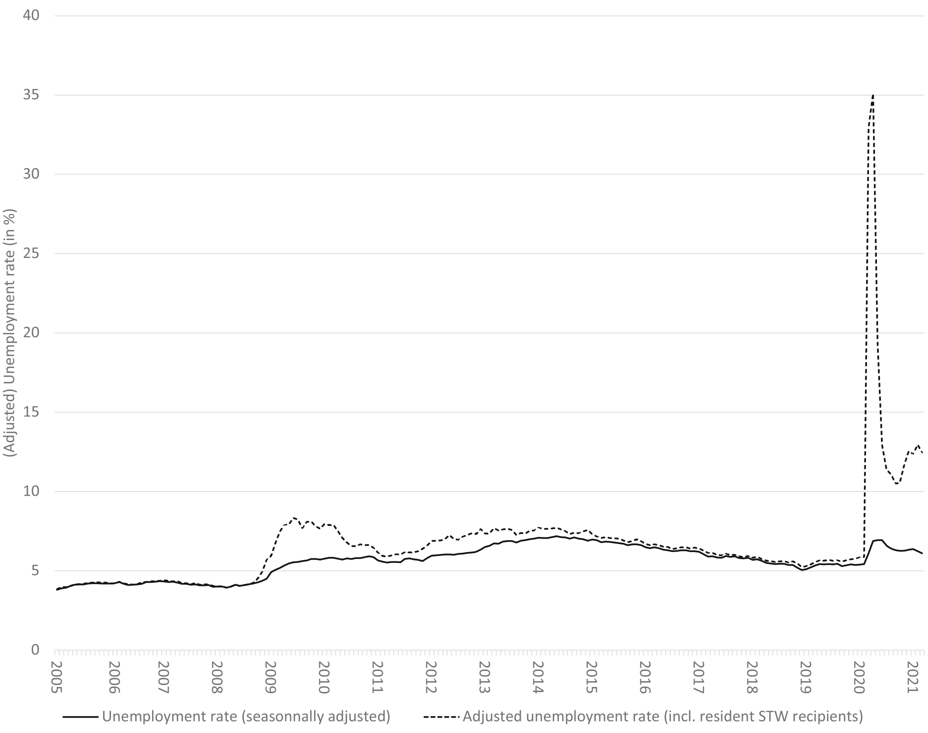

**Fig. 2** Evolution of the monthly unemployment rate, all industries. Note: Extracted from https://statistiques.public.lu/en/index.html on May 05, 2021. The numbers provided by Statec only include individuals who were registered as officially unemployed with the Agency for Employment Development ("Agence pour le développement de l'emploi"). The adjusted unemployment rate shows individuals on the short-time work scheme and those formally unemployed. The data on individuals on short-time work was provided by Statec upon request were on short-time work in April 2020. This number decreased following the gradual de-confinement and reached 5.5 percent in Q3. The highest shares of employees on the short-time work in April 2020 were in the construction, hospitality, and wholesale and retail trade sectors (87.0, 87.9, and 52.8 percent). These sectors were forced to shut down following the restrictions introduced in March as there are limited possibilities for individuals employed in these sectors to work from home (Dingel and Neiman 2020). In contrast, there was no short-time work in the public administration sector and it was extremely limited in other branches of the service sector (i.e. financial services, education, health, and social work). Activity in the majority of the sectors resumed in Q3 and Q4, with the exception of the hospitality sector, where the percentage of employees on short-time work remained high in Q3 and Q4 (31.1 and 40.8 percent). Table A-1 in the Supplementary Material provides a detailed overview of the share of workers on short-time work by industry and the average share of total hours on short-time work by industry for April, Q2, Q3 and Q4 2020.

In order to cushion the drops in labour incomes related to changes in employment the government introduced a set of new policy instruments, which complemented the existing tax-benefit system (see Table A-2 in the Supplementary Material). The largest, and probably the most influential, instrument for preserving formal employment in affected businesses was the short-time working scheme. For individuals that lost all their employment during the COVID-19 crisis a simplified procedure to access the normal unemployment benefit was put in place in order to reduces the frequency of physical contact.[6] The main differences between the two schemes are (EUROMOD 2020):

– different eligibility conditions (all employees are eligible for short-time work, which is not the case for the regular unemployment benefit scheme, where eligibility relies on the duration of employment prior to losing the job, country of residence, etc.);
– varying replacement rates depending on the family situation (the replacement rate for all employees under the short-time work is 80%, subject to a maximum, while the regular unemployment benefit may be increased to 85% if the dependent children are present in the household), subject to the same maximum;
– delay in payment of up to 2 months for the regular unemployment scheme due to the application procedure which is not the case for beneficiaries of short-time work (short-time work payments are processed through the employers' payroll system).

Apart from short-time work, the government also introduced a special family leave scheme. Between March and May, employees who had to interrupt their work in order to care for children below 13 could have benefited from a 100% salary replacement. The leave was prolonged beyond May for those employees whose children did not receive a place in childcare / educational facilities due to additionally imposed sanitary restrictions or who had to go on quarantine due to children's COVID-19 exposure. In the case where employees or their children tested positive to COVID-19, the National Health Fund covered the full salary cost.

Between June and December 2020, the government also announced an increase in the special allowance for low-income households lifting it from 110 Euros to 220 Euros per month. Finally, all individuals of 16 years and older residing or working in Luxembourg received a 50 Euro voucher to be used for an overnight stay in a hotel or other accommodation in Luxembourg. Table A-2 in the Supplementary Material provides a detailed description of all policies introduced in the context of COVID-19 and the main pre-existing automatic stabilizers.

## 3 Method: nowcasting and its need in times of crisis

In order to understand how economic and policy changes affect individuals and households, household survey data is usually used. There is, however, a time lag between its collection and release for research purposes. In Europe, the main survey with information on the income situation of households is the European Union Statistics on Income and Living Conditions (EU-SILC). The most recent wave of this survey dates back to 2018 and contains incomes for 2017 (the income reference year is always the year preceding the data collection year). Hence, one can expect that data for 2021 with incomes for 2020 will be available only in 2023. In normal times, substantial changes may occur over a two-year period between the moment of data collection and its availability for researchers. In a crisis, changes in the income situation of households are often so significant that a time lag can make data relatively meaningless, especially for a timely policy response.

[6]They are required to fill in a form supplied by the Luxembourg National Employment Agency (ADEM) and ADEM will then contact them to complete the application (Application has to be submitted within 2 weeks following the change in the employment situation). There is no more need to go to ADEM in person. The payment is received at the earliest on the 11th working day following the date of submission and at latest on the third last working day of the following month +2 to 3 days to receive the payment in the bank account.

There are other more recent data sources that can assist in the analysis of changes in the welfare of individuals and their households, such as the Labour Force Survey (available on a quarterly basis with a six-week lag) or up-to-date register and price data (available on a monthly basis with a short lag). However, these datasets do not contain household income information. For example, the main objective of the Labour Force Survey is to document the employment situation of individuals and their labour market incomes. Administrative data often contains detailed information on individual incomes from different sources but it does not always cover all possible sources of income or allow linking individuals within households. Another limitation of administrative data is the lack of information on socio-economic characteristics of individuals and their households. Understanding these characteristics is important for the development of targeted policies, which can help to mitigate the impacts of the crisis on the most vulnerable groups.

We overcome this data gap by applying a "nowcasting" methodology based on microsimulation (O'Donoghue and Loughrey 2014) using the most up-to-date data on employment, wage and price levels to calibrate a simulation model of household incomes, taxes and benefits following O'Donoghue et al. (2020) and O'Donoghue and Sologon (2021). This allows us to produce a "near real" time picture of the population and identify those that are most affected.

Most of the existing "nowcasting" methods are relatively crude, relying on price inflation factors and proportional changes of industry-specific employment rates combined with a tax-benefit model to explain the policy consequences (Navicke et al. 2014). This approach was applied in a handful of European countries to provide early scenario analysis assessing the impact of the COVID-19 crisis. This type of distributional transformation approach may sometimes bias the estimates if the impact of the shock is not uniformly distributed within the population. Bruckmeier et al. (2021) combine different data sources to estimate output shocks at the industry-level in real-time, a labour demand model to determine employment changes by industry and worker groups, and the microsimulation model to evaluate the distributional consequences. Their analysis undertakes a three-stage simulation determining who is affected, whether they become unemployed and finally the decrease in work hours. Within the input-industry cells, however, workers are assumed to be affected with a uniform distribution. An alternative method, as adopted by Li et al. (2020), is to combine several datasets via a semi-parametric re-weighting process to reflect the changes in the distribution. It follows the principle outlined in DiNardo et al. (1996) by adjusting the weights based on selected key covariates and further adjusts the income variable with external statistics. Their approach, however, requires the availability of the actual labour market distribution both immediately before and after the shock, which is not available in the case of Luxembourg. A reweighting methodology based on economic forecasts for 2020 on GDP and employment coupled with the EUROMOD tax-benefit model was applied by Almeida et al. (2021) in a comparative study at the EU level. As underlined by Canto et al. (2021), the reweighting approach has several drawbacks, one notable being it ignores heterogeneous effect across the income distribution, which is overcome in a dynamic microsimulation setting.

We propose a more nuanced approach that allows us to capture the heterogeneity of changes in the population by utilizing a dynamic income generation model to update the data (Bourguignon et al. 2001; Li and O'Donoghue 2014). This approach relies on the generic household income generation model (IGM) developed by Sologon et al. (2021) to simulate the labour market situation and household market income distribution as a function of personal and household attributes and to generate counterfactual distributions under alternative scenarios.[7] In this paper, we extend the IGM to simulate counterfactual "near real-time"[8] income distributions as a function of more timely data in order to assess the distributional impact of COVID-19. In order to apply the dynamic nowcasting methodology, we require different data sources, which are discussed in detail in Section 4: calibration data to index income growth; calibration data to align the labour market; and microdata on which to estimate and simulate.

Thus, underpinning our study is a set of calibration control totals reflecting the changes in the macro-economic climate in Luxembourg over the period of the outbreak, particularly in relation to the structure of the labour market. We build up the micro data to the present and we calibrate the IGM developed in Sologon et al. (2021) data to external control totals (macro trends). This assures that the IGM is describing the most current data. We introduce various shocks (e.g. factoring sector-specific impacts, differentiated by age and gender, fiscal responses) and create counterfactual distributions under alternative scenarios. We assume no second order behavioural responses to the crisis (e.g. no labour supply responses due to the fear to be contaminated at the workplace or of the COVID-specific transfer policy interventions). We assume the changes in labour market participation are channelled through the shocks in the employment opportunities and wages rather than the presence of the virus per se.

To sum up, our approach relies on two core ingredients, described below:[9]

- a household income generation model to describe the overall household income distribution and create counterfactual distributions;
- a nowcasting component to calibrate the simulation from the income generation model to external statistics with the objective to provide a "near real-time" picture of the distribution of income.

## 3.1 Income generation model

The income generation model follows the approach developed in Sologon et al. (2021).[10]

### 3.1.1 Components of household disposable income

Household disposable income ($y_h$) is formed of 5 broad components:

$$y_h = \underbrace{y_h^L + y_h^K + y_h^O}_{Market} + \underbrace{y_h^B - y_h^T}_{Non-market} \tag{1}$$

where $y_h^L$ is gross labour income (including employee, self-employed incomes), $y_h^K$ is household capital income (including capital, rental incomes), $y_h^O$ is other household non-benefit pre-tax incomes (including private pension, private transfers, and other incomes), $y_h^B$ is public benefits, and $y_h^T$ is direct taxes and social insurance contribution. These broad components aggregate subcomponents of household incomes, which are modelled separately to

[7]We have previously used the income generation model for cross-country analysis (Sologon et al. 2021) and for historical analysis in Lithuania (Cerniauskas et al. 2021).

[8]As we align our simulation to the public release of the Labour Force Survey which occurs by quarters, we provide estimates with a lag of one quarter.

[9]The diagram in Fig. B-1 summarises the philosophy of the model.

[10]Please refer to the source methodology in Sologon et al. (2021) for a detailed discussion of the income generation model.

achieve a high level of disaggregation. Gross household labour income $y_h^L$ is composed of employment and self-employment income of all household members ($hi$).

$$y_h^L = \sum_{i=1}^{n_h} I_{hi}^{lab}(I_{hi}^{employed} y_{hi}^{employed} + I_{hi}^{selfemp} y_{hi}^{selfemp}) \quad (2)$$

$I_{hi}^{lab}$ is a binary indicator equal to 1 if the individual is working, and zero otherwise.Those working are classified into employed and self-employed by the binary indicators $I_{hi}^{employed}$ and $I_{hi}^{selfemp}$. For those employed, $y_{hi}^{employed}$ marks the level of employment income (wage*hours of work). For the self-employed, $y_{hi}^{selfemp}$ marks the level of self-employment income.

Capital income is composed of investment and property income. $I_{hi}^{inv}$ and $I_{hi}^{prop}$ are binary indicators equal to 1 in the person has investment/property income, whereas the level is represented by $y_{hi}^{inv}$ and $y_{hi}^{prop}$. Similarly, for the other income sources we have an indicator for the prevalence of the income source and their levels.

$$y_h^K = \sum_{i=1}^{n_h}(I_{hi}^{inv} y_{hi}^{inv} + I_{hi}^{prop} y_{hi}^{prop});\ y_h^O = \sum_{i=1}^{n_h}(I_{hi}^{pripen} y_{hi}^{pripen} + I_{hi}^{Other} y_{hi}^{Other}) \quad (3)$$

Household benefits ($y_h^B$) are defined as the sum of household pension income, means-tested benefits and non-means tested benefit, whereas direct taxes ($y_h^T$) are defined as a combination of income taxes and social security contributions (ssc):

$$y_h^B = y_h^{pens} + y_h^{mtb} + y_h^{nmtb};\ y_h^T = y_h^{tax} + \sum_{i=1}^{n_h} y_{hi}^{ssc}. \quad (4)$$

### 3.1.2 Modelling of income components

We specify parametric relationships between observed individual and household characteristics and each income source above. We follow closely the notations and approach in Sologon et al. (2021) for consistency.[11] For each income source, we first estimate the prevalence of the income source, $I_{hi}$, equal to one if the individual receives the income source, and zero otherwise. For those receiving it, we estimate the level $y_{hi}$.

For labour income, we first estimate the probability to be at work (to receive any income from work) conditional on individual characteristics $x_{hi}$. This is the indicator $I_{hi}^{lab}$ for person $i$ in household $h$ above. All our binary outcomes are estimated using logistic models (see Agresti 2010). The participation indicator for person $hi$ depends on her individual characteristics $x_{hi}$ and her random residual $\epsilon_{hi}^{lab}$ given model parameters $\Upsilon^{lab}$. Participation is estimated separately for men, single women and women in a couple.

Second we estimate the probability to be an employee versus a self-employed, conditional on being in work. The employed vs. self-employed indicators are determined by individual characteristics $x_{hi}$ and the random residual $\epsilon_{hi}^{emp}$ given model parameters $\Upsilon^{emp}$.

Third, for those in work, we model their occupation (8 categories, based on the ISCO-08 classification), industry [8 categories[12]]) and sector (private and public). Occupation

[11] See Sologon et al. (2021), Supplementary Material A for a detailed description. The structure and the estimation of the models in the IGM follows closely the original paper.

[12] Agriculture = A - Agriculture, Forestry and Fishing; Manufacturing = B - Mining and Querrying & C - Manufacturing & D - Electricity, gas, steam and air conditioning supply & E - Water supply, sewerage, and industry are modelled with a multinomial logistic model as Sologon et al. (2021) and Bourguignon et al. (2008) do. Occupation is determined by $x_{hi}$ and a set of 8 individual-specific extreme value distributed residuals $\epsilon_{hi}^{k,occ}(k = 1, ..., 8)$ given the model parameters $\delta^{occ}$determining the probability distribution of potential occupations.

Similarly, industry is determined by $x_{hi}$ and a set of 8 individual-specific extreme value distributed residuals $\epsilon_{hi}^{k,ind}(k = 1, ...., 8)$ given the model parameters $\delta^{ind}$.

Public sector is modelled with a binary logistic model and is determined by ($x_{hi}$, $\epsilon_{hi}^{pub}$, $\Upsilon^{pub}$). Public sector enters as conditioning variable in the model for occupation, which then enters as conditioning variable in the model for industry.

Income from self-employment is estimated by a log-linear regression model $y_{hi}^{se} = \exp(x_{hi}\beta^{se} + \upsilon_{hi}^{se})$, conditional on being self-employed. Income from salaried employment is given by $y_{hi}^{emp} = w_{hi} * s_{hi}$. $s_{hi}$ is the total number of hours of employment, which is projected onto $x_{hi}$ using a linear model. $w_{hi}$ is the average (hourly) wage rate for person $hi$. Given the central role of wages in the composition of household income, we use a flexible parametric distributional regression for wages which connects individual characteristics to the entire conditional wage distribution and not only the conditional mean (see Biewen and Jenkins (2005) and Van Kerm (2013)). Individual wage is given by $w_{hi} = F_{X=z}^{-1}(\upsilon_{hi}^{emp}) = b(z)[(1 - \upsilon_{hi}^{emp})^{-\frac{1}{q(z)}} - 1]^{\frac{1}{a(z)}}$, where $\upsilon_{hi}^{emp}$ is a random term uniformly distributed, $q(z)$ is a shape parameter for the 'upper tail', $a(z)$ is a shape parameter for the 'spread' affecting both tails of the distribution, and $b(z)$ is a scale parameter. $a$, $b$ and $q$ parameters depend log-linearly on individual characteristics ($z$) which include labour market characteristics (e.g. occupation, industry, sector).

To sum up, household labour income $y_h^L$ is characterised by the characteristics $x_{hi}$ of the $n_h$ household members, residual heterogeneity terms ($\epsilon_{hi}^{lab}$, $\epsilon_{hi}^{emp}$, $\epsilon_{hi}^{k,occ}$, $\epsilon_{hi}^{k,ind}$, $\epsilon_{hi}^{pub}$, $\epsilon_{hi}^{hrs}$, $\upsilon_{hi}^{se}$, $\upsilon_{hi}^{emp}$) given the model parameters ($\gamma^{lab}$, $\gamma^{emp}$, $\delta^{pub}$, $\delta^{\text{occ}}$, $\delta^{ind}$, ($\beta^{se}$, $\sigma^{se}$), $\gamma^{hrs}$, ($\beta^{a,emp}$, $\beta^{b,emp}$, $\beta^{q,emp}$)).

The other non-tax-benefit incomes are modelled at a lesser granular level as too few households hold them.The modelling follow the same logic. We estimate the prevalence of the income source, $I_{hi}^S$, using logistic models. For those receiving it, we estimate the level of the income source, $y_{hi}^S$, $S \in$ {inv, prop, pripen, other} using log-linear models. Household capital and other incomes, $y_h^K$ and $y_h^O$, are characterised by the characteristics $x_{hi}$ of the $n_h$ household members, residual heterogeneity terms ($\epsilon_{hi}^{inv}$, $\epsilon_{hi}^{prop}\epsilon_{hi}^{pripen}$, $\epsilon_{hi}^{other}$, $\upsilon_{hi}^{inv}$, $\upsilon_{hi}^{prop}$, $\upsilon_{hi}^{pripen}$, $\upsilon_{hi}^{other}$) given the model parameters.

These parametric relationships are reduced-form projections that describe the empirical associations between the household and individual characteristics and the income components. We use these estimated projections to simulate counterfactual distributions of disposable income under alternative scenarios.

waste managment and remediation activities; Construction = F - Construction; Commerce = G - Wholesale and retail trade; repair of motor vehicles and motorcycles & I - Accommodation and food service activities & J - Information and communication & K - Financial and insurance activities & L - Real estate activities & M - Professional, scientific and technical activities & N - Administrative and support service activities; Transport = I - Transporting and storage; Public Administration = O - Public administration and defence; compulsory social security; Education/Health/Social = P - Education & Q - Human health activities; Other = R - Arts, entertainment and recreation & S - Other services activities & Activities of households as employers; undifferentiated goods - and services - producing activities of households for own use.

Non-market income components resulting from public policy such as income taxes, social insurance contributions, social assistance benefits, social insurance benefits, and universal benefits are simulated using the EUROMOD tax-benefit microsimulation model for Luxembourg (see Sutherland and Figari 2013). Since the model encompasses present and historic tax-benefit policies, EUROMOD allows a user to swap policies between different periods (see for e.g. Levy et al. (2007), Bargain and Callan (2010) and Bargain 2012).[13]

### 3.2 Simulating counterfactual distributions and nowcasting

In a nutshell, the income generation model (IGM) relies on a system of hierarchically structured, multiple equation models for detailed income sources, combining:

- a set of personal (individual and household) characteristics, X,
- parameters describing how employment, the receipt and level of income sources vary with personal characteristics, $\xi$,
- residuals linking model predictions to observed income sources, $\Upsilon$, and
- a tax-benefit simulator for converting market incomes into disposable incomes - EUROMOD.

In our analysis, we use the latest available EU-SILC survey year ($s = 2017$) to estimate the parameters of the IGM. At time $s$, the IGM describing the distribution of disposable income can be formalized as

$$Y_s = m(X, \Upsilon; l_s(\xi); r_s(\xi); tb_s(\xi)), \tag{5}$$

where $Y$ is disposable income, $l$ is the labour market structure, $r$ is the structure of returns and $tb$ is the tax-benefit system. In other words, the IGM is a statistical representation of the structure of the presence and the level of market incomes (and its components), and the tax-benefit rules describing policy incomes. This structure could be used to simulate counterfactual distributions of disposable incomes under different scenarios. For example, simulating a different labour market structure (e.g. with regard to employment, occupation, industry) would affect not only the simulation of the labour market status of each individual, but also their income via various channels, such as wage changes triggered by changes in employment characteristics, benefits, taxes.

#### 3.2.1 Nowcasting the distribution to $t$ (2020 pre-COVID) and $t + 1$ (2020 during Covid)

The first step is to "nowcast" the most recent available survey data (period $s$) to reflect the situation in both periods of interest $t$ (2020 pre-COVID) and $t + 1$ (2020 during COVID). We follow the nowcasting methodology described in O'Donoghue and Sologon (2021) and applied in O'Donoghue et al. (2020).

We utilize the estimated IGM based on year $s$ described above to capture the relationship between the income components and demographic and labour market characteristics. We simulate the changes in the distribution of disposable income by calibrating the labour market, income and tax-benefit transformations so as to reflect the situation in period $t$ and $t + 1$.

[13]We use regression techniques to model the distribution of the partially simulated and non-simulated variables as described in Sologon et al. (2021). A summary of the variables modelled by EUROMOD and by regression models is available in Supplementary Material Tables C-3 and C-5.

We outline below the transformations in a general form and leave the exact variables on which the transformations are applied to the Supplementary Material Tables C-3, C-4 and C-5. We perform three 'transformations'.

(1) The *labour market structure transformation* involves simulating the labour market structure and changing important characteristics of the labour market structure such as employment, occupation and industry. This involves using the parameter estimates and the residuals of the labour market models of period $s$ to simulate the labour market structure and calibrating these simulations to the external labour market statistics. The labour market calibration uses two types of alignment: for discrete binary data and for discrete data with multiple choices. For example, we simulate first who is in work, calibrating it by external statistics with respect to the in-work composition by age and gender. We select on the highest rank of the latent variable, such that we obtain the numbers in the calibration totals. In doing this we draw upon the dynamic microsimulation literature and overcome a series of simulation issues present in other calibration approaches Li and O'Donoghue (2014). For those simulated as in work, we simulate next whether they are employees, their occupation and industry choices, again calibrated to match external labour market statistics. In multiple-choice models (e.g. occupation, industry), we apply a similar calibration technique: we rank the latent variable for each choice $j$, and we select such that to be consistent with external statistics for each category (e.g. occupation and industry structure). Using the rest of the models describing the labour market, we simulate all the remaining characteristics. To sum up, this step involves simulating who is in-work, who is an employee/self-employed, occupation, industry, sector, who is in short-time work, who is out-of- work, who is unemployed, etc., in order to reflect external labour market statistics. In our study, we rank by a probability that accounts for heterogeneity of employability plus a random term, calibrated within industry categories, and not by a uniform number as done in most studies.

This results in a labour market structure aligned with the external statistics with respect to the in-work composition by age and gender, the composition of employment/self-employment by gender, the occupation and industry structure by gender, the unemployment structure by gender. All individuals in the working age group will have an updated labour market status and updated labour market characteristics, captured by the new vector $\tilde{l}_t(\xi)$, which reflects the labour market structure at time $t$.

(2) The *returns transformation* involves two steps. First, we simulate all income sources based on the new labour market structure simulated in the previous step using the estimated parameters of the IGM. For example, the wage model will simulate wages for all individuals to capture potential changes in employment, occupation, industry, sector, etc. Those who lose their jobs or transition into short-time work have their incomes simulated accordingly.

Second, we update all income sources applying the EUROMOD uprating factors for all the pre-fiscal monetary variables to align them with the policy parameters of the targeted year (to be applied in the next step). For our simulations, pre-fiscal monetary variables from period $s$ need to be inflated to the year of the tax-benefit system being considered (period $t$) by using the EUROMOD uprating indices embedded in the system. This is needed to align pre-fiscal incomes with the tax-benefit parameters both in terms of productivity and prices. EUROMOD applies a vector of uprating factors: for example employment income, wages, some benefits (e.g. unemployment, maternity, social assistance) are uprated by the annual average minimum wage index; several income components, such as self-employment income, investment income, private pensions, private transfers, and some benefit monetary variables are uprated by the national consumer price index; some benefit monetary variables are uprated by benefit specific indices (for example, old age benefits).This results in an updated pre-fiscal income vector for all individuals, $\tilde{r}_t(\xi)$, expressed in period $t$ values.

(3) The *tax-benefit system transformation* updates the tax-benefit rules to reflect the nowcasted period, simulated by EUROMOD, to produce an alternative parameter vector $\tilde{tb}_t(\xi)$. This involves using EUROMOD to apply the tax-benefit rules and parameters of the nowcasted period onto the market incomes and household characteristics updated in the previous steps. These simulations result in the nowcasted distributions of disposable income:

- before the crisis, $\tilde{Y}_t = m(X, \Upsilon; \tilde{l}_t(\xi); \tilde{r}_t(\xi); \tilde{tb}_t(\xi))$, and
- during the crisis, $\tilde{Y}_{t+1} = m(X, \Upsilon; \tilde{l}_{t+1}(\xi); \tilde{r}_{t+1}(\xi); \tilde{tb}_{t+1}(\xi))$.

The change in the distribution of disposable income under the impact of the crisis is assessed by taking the difference $\tilde{Y}_{t+1} - \tilde{Y}_t$, measured using a distribution functional of interest, $\theta$, such as the Gini index or the quantiles.

$$\Delta_\theta(F) = \theta(F_{t+1}) - \theta(F_t). \tag{6}$$

### 3.2.2 Simulating alternative scenarios to decompose the distributional changes between *t* and *t* + 1

We are interested next in separating the effect of the labour market shock from the effect of the policy response. This is done with the help of counterfactual distributions.

In order to identify the labour market shock we contrast the distribution at time $t$ with the counterfactual distribution that would prevail if in period $t$ we "import" the labour market shock of period $t + 1$, keeping everything else the same. This is obtained by performing a labour market transformation of period $t$ to reflect the labour market structure and the shock of period $t + 1$ and resimulating incomes accordingly, while maintaining the uprating factor for monetary variables in line with the policy parameters at period $t$. The effect is represented formally by:

$$m(X, \Upsilon; \tilde{l}_{t+1}(\xi); \tilde{r}_t(\xi); \tilde{tb}_t(\xi)) - m(X, \Upsilon; \tilde{l}_t(\xi); \tilde{r}_t(\xi); \tilde{tb}_t(\xi)). \tag{7}$$

In essence, this is a counterfactual situation in which we have the COVID shock, but no COVID-specific policy intervention and the "old" system is allowed to buffer the shock via its automatic stabilizers.

In order to identify the distributional effects of the COVID-specific policy intervention, we contrast the distribution at time $t+1$ which incorporates the labour market shock and the COVID-specific policy response with the counterfactual distribution of no COVID-specific policy intervention:

$$m(X, \Upsilon; \tilde{l}_{t+1}(\xi); \tilde{r}_{t+1}(\xi); \tilde{tb}_{t+1}(\xi)) - m(X, \Upsilon; \tilde{l}_{t+1}(\xi); \tilde{r}_t(\xi); \tilde{tb}_t(\xi)) \tag{8}$$

We compute the impact on the Gini index and the quantiles as follows:

$$\Delta_\theta(F) = (\theta(F_t^*) - \theta(F_t)) + (\theta(F_{t+1}) - \theta(F_t^k)) \tag{9}$$

where $\theta(F_t^*)$ is the distribution functional of interest in the scenario with the COVID labour market shock but without a COVID-specific policy intervention.

## 4 Data

As highlighted above, the application of the dynamic methodology requires two types of data: (i) microdata used for estimations and simulations, and (ii) calibration data used to align the microdata to macro trends reflecting changes in the labour market. The main source of the microdata is EU-SILC. The dataset contains detailed information on personal and household incomes, demographics and labour market characteristics of individuals, reflecting a nationally representative sample of the resident population. For our analysis, we have used the EUROMOD standardised datasets based on 2018 EU-SILC for Luxembourg, which are suitable for tax-benefit modelling.[14]

Our analysis is underpinned by a set of calibration control totals reflecting the changes in Luxembourg's macroeconomic situation that occurred since the collection of the EU-SILC data in 2018 and up to the current COVID-19 crisis. We use the calibration control totals to calibrate the income generation model in order to align the information in EU-SILC with the labour market situation during COVID-19. More precisely, we simulate the changes in the distribution of household incomes by calibrating the components of the IGM to reflect the labour market with regard to the employment situation and income levels by age, gender, industry and occupation, and tax-benefit transformations of the target period. The data used for the calibration is drawn from three main sources:

- Labour Force Survey;
- Administrative data;
- Survey on the Socio-Economic Impact of the COVID-19 Crisis (SEI).

The Labour Force Survey (LFS) is published by Eurostat and is available on a quarterly basis with a 6–8 week lag. Given the asymmetric nature of the COVID-19 crisis, our labour market alignment is differentiated by age, gender, occupation and industry. For this purpose, we have extracted information on the employment rates by age, gender, occupation and industry for Q4 2019 (in order to ensure that our pre-crisis estimates do not capture any impact of the crisis), and Q2 , Q3 and Q4 2020. In the analysis, we refer to pre-crisis period (reflecting Q4 2019) as Q1 to simplify the interpretation of the results. Q2, Q3 and Q4 refer to periods April-June, July-September and October-December 2020.

For the alignment of short-time work, we use monthly statistics on the number of approved short-time working claims by industry, collected by the Luxembourg National Employment Agency (ADEM) and provided by the Luxembourg National Statistical Office (STATEC):

(i) April - to capture the peak of the first wave, and
(ii) average monthly claims for Q2, Q3 and Q4 - to capture the development of the pandemic after the first COVID-19 wave.

As the data on the number of individuals under short-time work does not differentiate between residents and cross-border workers, we utilized administrative statistics collected by the General Inspectorate of Social Security (IGSS) and provided by STATEC to compute the share of residents in total employment by industry. The share of resident workers in total employment was then applied to the total number of workers under short-time work. We thereby assume that the likelihood of falling under short-time work does not differ between residents and cross-border workers employed within the same industry. IGSS data was also used to calibrate the number of retirees by gender, and the share of resident workers in the total workforce to reflect the situation in 2020.

[14]All benefits, except short-time work, are modelled in EUROMOD assuming full benefit take-up. In this paper, we prioritized the use of near real-time data over detailed modelling of benefit take-up. We however acknowledge the importance of benefit non-take-up and would explore the issue in future work.

To model the likelihood of individuals to be put under short-time work, we estimated a model utilizing the survey data on the Socio-Economic Impact of the COVID-19 Crisis (SEI) collected during the pandemic.[15] This model includes a range of demographic characteristics, such as age, household composition, marital status and education, but also information regarding the individual's occupation and industry of employment.

In order to assess the distributional impact of the COVID-19 crisis, we compare the nowcasted distribution of income in the first quarter of 2020 (reflecting the labour market situation in Q4 2019, before the crisis) with the nowcasted distributions for (i) April (at the peak of the first wave), (ii) Q2, (iii) Q3 and (iv) Q4. The disposable income concept used is the equivalized household disposable income. We equivalize the standard EUROMOD definition of disposable income by dividing total household income by the square root of the household size (LIS equivalence scale).[16] The population composition and the aligned labour market structure based on the LFS, SEI and administrative statistics are shown in Table D-6.

**Time period of analysis**

The EUROMOD Tax-Benefit model uses the EU-SILC dataset which has an annual period of analysis. For our analysis, we have converted annual incomes into average monthly incomes using the labour market information available in EU-SILC. However, given the volatility over the crisis, we transform the unit of analysis into current monthly income at the employment level of a particular month by calibrating our analysis to monthly employment data provided by the Labour Force Survey (LFS) and monthly data on the number of workers on short-time work provided by the national statistics office STATEC.

For example, the April 2020 scenario refers to the nowcasted average monthly income using the LFS labour market statistics for the second quarter (Q2) and the April national statistics for short-time work to capture the shock. We have used the LFS labour market statistics published in June 2020 (Q2) as representative for April to calibrate the labour market. As commonly known, the LFS employment statistics also register those under short-time work as employed, as they retain their affiliation with their employer. In order to correctly calibrate the labour market with respect to those in short-time work, we therefore used monthly administrative statistics regarding the share of workers under short-time work by industry. In our analysis, the April 2020 scenario assumes the shock observed in April persists for the entire year, and the reported result is the monthly average.

The Q2 2020 scenario refers to the nowcasted average monthly income using the LFS labour market statistics for Q2 and the average share of workers on short-time work across April-May-June (Q2). We thus capture the average labour market shock across Q2 using the LFS labour market statistics published in June 2020 (Q2) as representative for average monthly employment across all months in Q2. In order to calibrate the labour market to short-time work, we have use the administrative statistics on short-time work averaged across April-May-June. Similarly to April, the Q2 scenario assumes that the same situation persists for the whole year and the reported results are montly averages. The same applies to Q3 and Q4.

In short, April 2020 shows the distribution of average monthly equivalized disposable incomes under the shock captured by the April macro statistics; Q2, Q3, Q4 shows the

[15]SEI was collected by the Luxembourg Institute of Socio-Economic Research and the University of Luxembourg.

[16]The standard EUROMOD definition of disposable income refers to the definition implemented in the tax-benefit simulator. Household disposable income is formed of 5 broad components, as shown in Eq. 1.

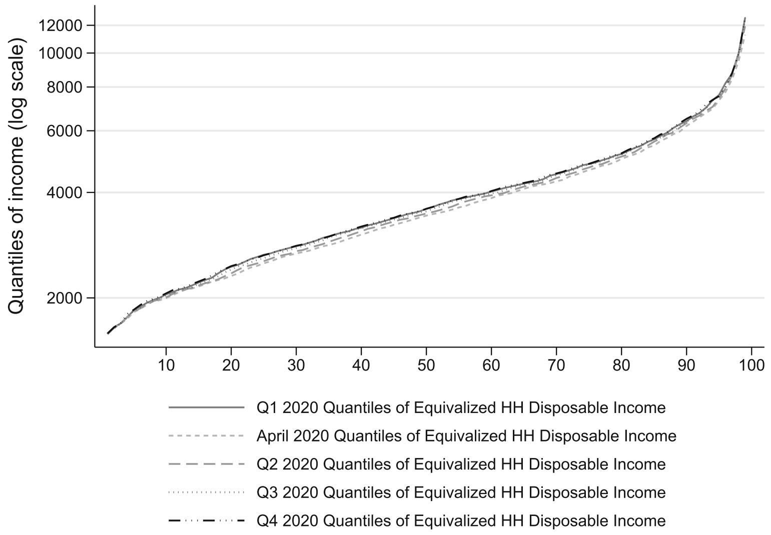

**Fig. 3** Pen Parades: 2020 Q1 vs 2020 April, Q2, Q3 and Q4 nowcasted distribution of average monthly equivalized disposable incomes under the shock captured by average Q2, Q3 and Q4 macro labour market statistics.

The Q1 2020 scenario refers to the nowcasted average monthly income using the LFS labour market statistics for pre-crisis. In order to avoid the contamination of the March figures, we use the LFS for Q4 2019 to reflect the situation pre-Covid.

## 5 Nowcasting results

### 5.1 Distributional changes before and during the COVID crisis

Figure 3 presents the evolution of the distribution of household disposable income in different periods of 2020 - before the COVID-19 outbreak (first quarter of 2020), at the peak of its first wave (in April), and during the second, third and forth quarters of 2020 - using the Pen's parades diagram.[17] On this diagram, all individuals are lined up according to the size of their monthly household disposable income from the poorest to the richest. Figure 3 shows that, before the crisis, monthly incomes of the poorest 10 percent did not exceed 2000 Euros per month whereas monthly incomes of the richest 10 percent were above 6000 Euros per month. Following the crisis outbreak, all individuals experienced a drop in disposable income. Income losses were the largest in April, when the country entered a strict lockdown, decreased in size following the lifting of the lockdown measures in Q2 and Q3, and came back to the pre-crisis level towards the end of 2020 (Q4).

In order to better reflect the changes in incomes at different stages of the crisis, we take the differences between the Pen's parades in various quarters of 2020 and Q1. Figure 4a reveals that, both at the peak of the crisis and right after it (in Q2), the absolute losses in

[17]Note that all the results presented in Section 5 refer to the total population (including those who do not work). In this context, stabilisation of income losses might also take place within households.

**Table 1** Equivalized household disposable income in Q1 2020, April 2020, Q2 2020, Q3 2020, Q4 2020 (average monthly, in euros)

| | Mean | Median | Gini |
|---|---|---|---|
| Q1 | 4037.5 (3987.6, 4088.8) | 3594.9 (3523.1, 3648.020) | 0.260 (0.254, 0.267) |
| April | 3878.2 (3828.5, 3926.6) | 3434.9 (3387.1, 3470.295) | 0.260 (0.254, 0.267) |
| Q2 | 3944.3 (3894.9, 3998.7) | 3483.2 (3438.4, 3520.185) | 0.261 (0.256, 0.268) |
| Q3 | 4009.5 (3958.5, 4061.1) | 3532.7 (3503.2, 3598.508) | 0.262 (0.255, 0.268) |
| Q4 | 4056.0 (4002.7, 4106.2) | 3591.7 (3531.5, 3643.480) | 0.260 (0.254, 0.267) |

Bootstrap CI in parentheses based on 500 iterations

Euros in household equivalized disposable income were the smallest at the bottom of the distribution and the largest at the top. In April, on average, the disposable income of the poorest 10 percent dropped by less than 50 Euros per month whereas the income of the richest 10 percent decreased by 300 Euros per month. Income losses induced by the crisis diminished in Q3 and disappeared towards the end of the year. In relative terms (Fig. 4b), the income losses across the distribution in April, Q2 and Q3 are U-shaped, indicating larger relative losses for the middle (between 20th and 70th percentiles), than for the bottom and the top of the distribution.

In order to relate these income drops to shifts in income inequality, in panel (c) of Fig. 4 we also report the changes in the normalized distributions obtained by dividing the quantile values by the mean of the distribution. This allows us to see the changes in incomes that relate directly to changes in income inequality.

The fluctuations are small (between −0.02/+0.02) during all stages of the crisis, indicating minor (if any) changes in disposable income inequality, as will be shown later in Table 1. The U-shape is maintained: relative to the mean, the poorest 15% were better off in April and Q2 than in Q1, whereas the middle became worse off and the top fluctuated around zero.

Table 1 summarizes changes in the distributions of household disposable income across the five nowcasted periods of 2020: Q1, April, Q2, Q3 and Q4. Both average and median equivalized household incomes decreased the most in April compared to Q1. Incomes show signs of recovery across quarters, reaching pre–crisis levels in Q3 and Q4, which do not differ significantly from Q1. The observed declines in individual incomes, however, have not translated into sizeable changes in income inequality, which remained stable throughout 2020, as measured by the Gini index.[18]

## 5.2 Changes in redistribution during the crisis

In this section, we shed further light on the role of taxes and benefits in mitigating the impact of the crisis on the distribution of household disposable income in Luxembourg. We start by exploring the change in the Gini as we make the transition from gross income without benefits to gross income with benefits and then inspecting how it changes once we account for taxes and social security contributions.[19]

[18]The changes in alternative inequality measures - mean log deviation (GE0) and Theil index (GE1) - show a similar trend and can be provided upon request.

[19]Supplementary Material E also provides additional evidence on the contribution of various income sources to the evolution of Gini coefficient during the COVID-19 crisis.

**Fig. 4** Absolute, relative and normalized nowcasted changes: 2020 Q1 vs 2020 (April, Q2, Q3 and Q4)

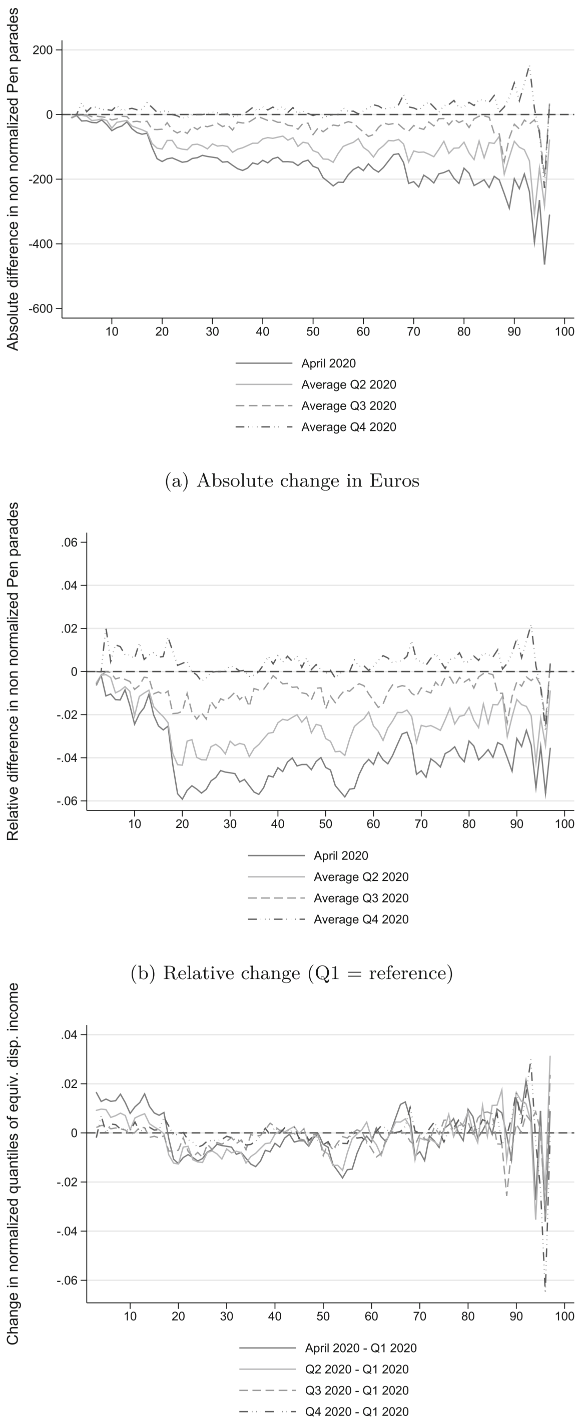

(a) Absolute change in Euros

(b) Relative change (Q1 = reference)

(c) Change in pen parades divided by their respective means

Table 2 presents the summary indicators of the overall effect of the tax-benefit system, as well as the sequential redistributive effects of benefits and taxes following Lambert (1992), evaluated at the peak of the crisis in April and the following quarters of 2020. The net redistributive effect of the tax-benefit systems is measured by the difference between the Gini of market income and the Gini of disposable income. For each component (benefits and taxes), we present the measures of (i) redistribution, given by the Reynolds-Smolensky index (difference in Ginis pre-post component); (ii) average tax (transfer) rates, defined as the ratio between the total amount of taxes (transfers) paid (received) and the total pre-tax (transfer) income; and (iii) progressivity (taxes)/regressivity(for benefits) effect, measured by the Kakwani index (Kakwani 1977).[20]

We find a sharp increase in inequality in gross incomes in April, followed by a gradual decline across quarters (with Q4 being more unequal than Q1). Adding benefits has a strong equalization effect on the distribution across quarters. Moreover, starting from very unequal market distributions, adding benefits results in similar levels of inequality across quarters. This is the result of a strong benefit redistribution at the peak of the crisis, which tapered off across quarters in response to the shock, as shown by Reynolds-Smolensky index. Benefits became more generous as reflected in the average transfer rate, which increased from 0.354 in Q1 to 0.591 in April 2020 and was still 8.6 percent higher at the end of the year compared to Q1. At the peak of the crisis, they also became less regressive implying that more people along the distribution were receiving benefits than before.

In contrast to benefits, the redistributive role of taxes remained unchanged throughout 2020. This is not surprising given that the benefits provided to compensate for losses in market incomes during the COVID-19 crisis had a high replacement rate and were taxed.[21]

Overall, Table 2 shows that net redistribution increased during the crisis and was driven mainly by the increase in benefit redistribution. In the following section we investigate whether redistribution increased as a result of the introduction of new policy instruments described in Table A-2 or of the labour market shock.

### 5.3 Contribution of the labour market shock and policy response to the changes in disposable incomes and inequality

When assessing the contribution of the labour market shock and policy response to the changes in disposable incomes and inequality, we report the situation at the peak of the

[20]The Kakwani index, calculated as the difference between the concentration coefficient of the given public intervention and the Gini coefficient before this intervention, can fluctuate between −1 and 1: negative values signal a disequalizing effect of the instrument on the income distribution and positive values signal an equalizing effect. Positive values of K for benefits indicate that benefits are regressively distributed, meaning that more transfers are allocated to lower income households. Thus they will exert an equalizing effect on the income distribution. The higher the K, the more regressive the benefit scheme. The average transfer rate shows the generosity of the benefit, whereas the degree of regressivity shows the extent to which benefits are concentrated in the lower part of the distribution i.e. who receives the transfer. They both contribute to benefit redistribution.

Positive values of K (up to 1) for taxes indicate that taxes are progressive, meaning that as we go up the ranks of the income distribution, the amount of taxes paid increases. Progressive taxes exert an equalizing effect on the income distribution. Please refer to Lambert (1992) for a detailed explanation of these indices.

[21]The classical unemployment scheme and the short-time work scheme are both subject to individual income tax. The same holds for the pandemic special family and sick leave. Since the replacement rates are extremely high for these schemes (See Table A-2), neither the average tax rate nor the tax progressivity changed significantly enough to produce changes in the degree of redistribution via taxes.

**Table 2** Progressivity and redistribution of taxes and benefits on household equivalized disposable income

| | Q1 | April | Q2 | Q3 | Q4 | Ratio: April/Q1 | Ratio: Q2/Q1 | Ratio: Q3/Q1 | Ratio: Q4/Q1 |
|---|---|---|---|---|---|---|---|---|---|
| Gini Gross Income | 0.484 (0.475, 0.494) | 0.571 (0.561, 0.581) | 0.538 (0.528, 0.547) | 0.503 (0.495, 0.512) | 0.499 (0.491, 0.509) | 1.179 | 1.110 | 1.039 | 1.031 |
| Gini Gross Income (+ benefits) | 0.328 (0.320, 0.336) | 0.328 (0.320, 0.336) | 0.330 (0.322, 0.338) | 0.331 (0.323, 0.339) | 0.329 (0.321, 0.337) | 1.000 | 1.005 | 1.008 | 1.002 |
| Average transfer rate | 0.354 (0.335, 0.374) | 0.591 (0.562, 0.623) | 0.482 (0.458, 0.507) | 0.390 (0.371, 0.412) | 0.385 (0.365, 0.406) | 1.669 | 1.361 | 1.101 | 1.086 |
| Benefit Regressivity (K) | 0.910 (0.893, 0.926) | 0.896 (0.882, 0.910) | 0.906 (0.892, 0.921) | 0.916 (0.900, 0.930) | 0.912 (0.897, 0.927) | 0.985 | 0.996 | 1.007 | 1.003 |
| Benefit Redistribution (RS) | 0.156 (0.149, 0.163) | 0.243 (0.235, 0.251) | 0.208 (0.201, 0.216) | 0.173 (0.166, 0.180) | 0.171 (0.164, 0.178) | 1.556 | 1.330 | 1.106 | 1.093 |
| Gini (gross + benefits - taxes) | 0.268 (0.262, 0.274) | 0.268 (0.262, 0.275) | 0.270 (0.264, 0.276) | 0.270 (0.264, 0.276) | 0.268 (0.262, 0.275) | 1.001 | 1.007 | 1.007 | 1.001 |
| Average tax rate | 0.165 (0.161, 0.168) | 0.160 (0.156, 0.164) | 0.162 (0.159, 0.166) | 0.165 (0.161, 0.168) | 0.166 (0.162, 0.170) | 0.971 | 0.987 | 1.000 | 1.008 |
| Tax Progressivity (K) | 0.314 (0.309, 0.320) | 0.322 (0.316, 0.328) | 0.319 (0.313, 0.324) | 0.317 (0.311, 0.322) | 0.313 (0.307, 0.319) | 1.024 | 1.013 | 1.007 | 0.995 |
| Tax Redistribution (RS) | 0.061 (0.059, 0.063) | 0.060 (0.058, 0.062) | 0.061 (0.059, 0.063) | 0.061 (0.059, 0.063) | 0.061 (0.059, 0.063) | 0.991 | 0.999 | 1.007 | 1.005 |
| Gini Disposable Income | 0.260 (0.254, 0.267) | 0.260 (0.254, 0.267) | 0.261 (0.256, 0.268) | 0.262 (0.255, 0.268) | 0.260 (0.254, 0.267) | 1.001 | 1.006 | 1.007 | 1.002 |
| Net Redistributive Effect | 0.224 (0.217, 0.232) | 0.311 (0.303, 0.319) | 0.276 (0.269, 0.284) | 0.242 (0.235, 0.249) | 0.239 (0.232, 0.246) | 1.385 | 1.230 | 1.077 | 1.066 |

Bootstrap CI in parentheses based on 500 iterations. K = Kakwani; RS = Reynolds-Smolensky crisis.[22] The changes in the income distribution can be decomposed into two main parts, with one being the change induced by the labour market shock (part A) and the other due to the public policy response (part B):

$$\begin{aligned}\Delta_\theta(F^{April}, F^{Q_1}) &= \theta(P^{April}, LM^{April}) - \theta(P^{Q_1}, LM^{Q_1}) = \\ &= \theta\,(P^{Q_1}, LM^{April}) - \theta(P^{Q_1}, LM^{Q_1})\,\{partA\} \\ &\quad +\theta(P^{April}, LM^{April}) - \theta(P^{Q_1}, LM^{Q_2})\,\{partB\}\end{aligned} \tag{10}$$

Q1 refers to the situation at the start of 2020, before the COVID crisis: $\theta(P^{Q_1}, LM^{Q_1})$. This calibrates our simulations to the labour market situation at the beginning of the year using the LFS. $P^{Q_1}$refers to the tax-benefit rules in place in the beginning of 2020, before the introduction of any COVID-specific policy interventions.

April refers to the peak of the crisis: $\theta(P^{April}, LM^{April})$. This calibrates our simulations to the labour market situation during the second quarter of 2020, whereas the labour market shock in terms of short-time work is aligned using monthly administrative statistics for April. We assume that all people who fall into short-time work reduce their hours by 100%. These people will retain the affiliation with their employer, but they will fall under the COVID compensation scheme. This is the base scenario for April and will provide lower bound estimates (a worst case scenario).[23]

$P^{April}$refers to the tax-benefit rules in place during the crisis period, which include the policy rules from Q1 and the COVID-specific policy interventions described in Table A-2. We use the policy rules implemented in EUROMOD, which cover mainly the short-time work scheme. The special family and sick leave for COVID-related issues offer full-insurance, providing 100% replacement of lost income, and thus would not affect the distribution of disposable income.[24]

The scenario of "No COVID policy intervention" is obtained by applying the pre-COVID policy to the labour market shock in April: $\theta\ (P^{Q_1}, LM^{April})$. We assume that all subsidised jobs would be lost should there be no compensation scheme[25]. This will serve as our counterfactual for no COVID policy intervention. Contrasting this counterfactual with the actual situation in Q1 (before the crisis), $\theta(P^{Q_1}, LM^{Q_1})$, we obtain the impact of the COVID labour market shock (Part A) on the distribution of income (be it the entire distribution or a particular index).

Contrasting the actual situation in April with the counterfactual in April under the "No COVID policy intervention" scenario, $\theta(P^{April}, LM^{April}) - \theta(P^{Q_1}, LM^{April})$, we obtain the policy effect on the distributional changes before/during the crisis.

[22]Supplementary Material F reports also the decompostion for Q2. The findings are consistent across periods, albeit with reduced effects for Q2 compared to April.

[23]Alternatively, we could also align the reduction of hours worked under the compensation scheme based on national external statistics.The results for this scenario are consistent with the results for April, but as expected are smaller in magnitude. We did not include them in the paper, but they can be provide upon request.

[24]Future work will try to model the infection rate and special family leave take-up, taking into account industry differentials. This would however not affect the distribution of disposable income, but the degree of benefit redistribution, which would be higher.

[25]There is likely some deadweight loss associated with STW. In modelling the policy response, we prioritized the use of near real-time data over detailed modelling of short-time work take-up. The assumption that all subsidised jobs would be lost in the absence of the STW scheme therefore provides an upper-bound scenario. Deadweight losses are however an important aspect of the schemes functioing, particularly when it is evaluated in relation to counterfactual scenarios. Detailed modelling of STW would be of interest for future research.

In Fig. 5, we identify the contribution of the labour market shock and of the policy intervention to the changes in the distribution of disposable income at the peak of the COVID-19 crisis observed in April. The upper panels highlight the decomposition results for the absolute nowcasted changes in the quantiles of equivalised household disposable income, whereas the lower panels show the decomposition results for the relative changes in the quantiles (April-Q1)/Q1). Figure F-5 in the Supplementary Material provides the results of the similar decomposition performed for Q2.

Most of the change in the distribution is explained by the labour market shock, which follows closely the observed change: assuming no COVID policy intervention, under the labour market shock, people lost income throughout the distribution. Both in absolute and relative terms, the bottom 20% lost less than the rest of the distribution. The scenario at the peak of the crisis (in April) shows a consistent story with the average scenario across Q2 (See Supplementary Material F), only with a more pronounced effect.[26]

The effect of changes in the tax-benefit rules during the crisis fluctuates around zero across the distribution, which indicates that the system was well equipped ahead of the crisis to cushion household incomes against job losses. In essence, there is little difference in the income compensation between the pre-existent unemployment benefit scheme and the short-time work scheme introduced in response to the COVID-19 pandemic. The main difference lies in the speed with which individuals access cash-flow support under the two schemes and the additional eligibility conditions for the existing unemployment scheme.[27] Under the new COVID scheme, people who fell into short-time work could access benefits faster than via the standard unemployment scheme while remaining formally employed.

Comparing the absolute level of income replacement to the relative level of income replacement, it becomes clear that although individuals in the bottom half of the income distribution benefited from higher absolute levels of income replacement, the relative level of income replacement was similar for individuals throughout the distribution of incomes.[28] A potential explanation for this pattern lies in the design of the benefits (which are earnings-related rather than means-tested) and in the fact that policy action may be more feasible politically if those affected perceive similar levels of relative income replacement.

When we move from the detailed distribution to the decomposition of the Gini index in Table 3, we find that under the labour market shock, the distribution of market incomes became more unequal (by 8.7 Gini points in April), the effect however is cancelled by the tax benefit intervention (see Table F-8 in the Supplementary Material for Q2). The zero

[26]For brevity reasons, we do not present the results for Q3 and Q4 because the trend continued over the year and the situation returned to the pre-crisis level in Q4.

[27]One should keep in mind that except of compensating for the loss of labour incomes the short-time work scheme also has other labor market effects as compared to standard unemployment benefits. One of the main objectives of the short-time work (STW) scheme is to provide employers with flexibility in managing working hours of their employees without turning to layoffs if those hours have to be reduced due to unfavourable business situations. By maintaining the employment link, STW supports employers in preserving their human capital and minimizes recruitment costs in the future. From the workers' side, they remain legally employed and can restart working as soon as the economic situation improves without searching for a new job.

[28]Albeit relative income losses were quite similar along the distribution of household disposable incomes, it does not imply that low-income and high-income individuals also experienced a relatively similar decline in their living standards. In fact, as shown for Ireland by O'Donoghue et al. (2020), a decline in housing and work-related expenses during the pandemic resulted in the strengthening of purchasing power of individuals in the lower tail of the distribution to a larger extent than those at the top. As a result, low-income individuals could afford buying more products and services than before. Contrarily, consumption constrains induced by the pandemic (e.g. travel restrictions, restaurant closures) affected high-income households more than low-income households (Coffey et al. 2020) reducing their living standards.

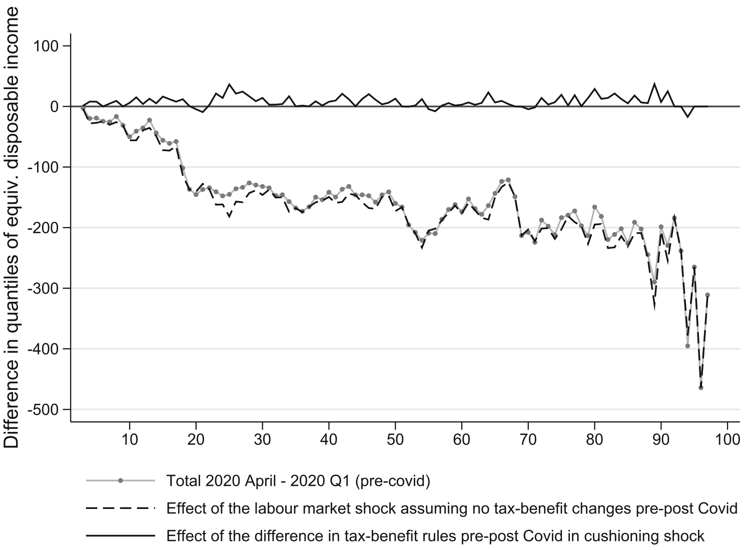

(a) Absolute decomposition: April

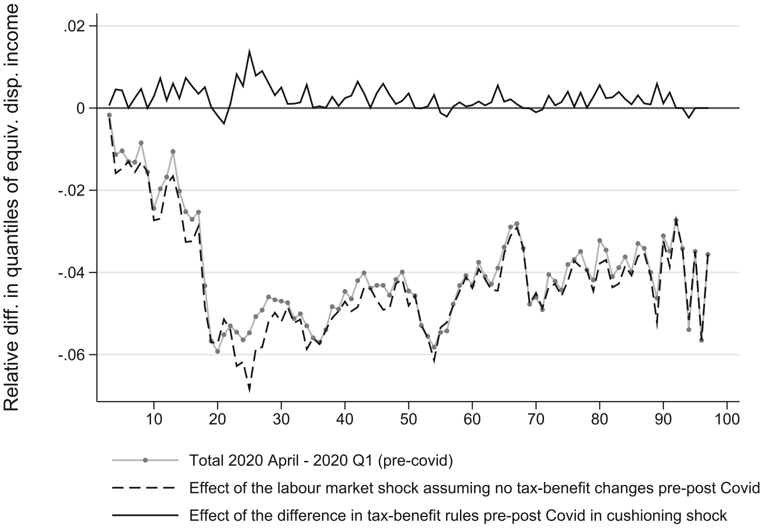

(b) Relative decomposition: April

**Fig. 5** Contribution of the labour market shock and policy response to the change in income centiles under the COVID crisis: April-Q1

effect of the changes in the tax-benefit rules judged against the COVID shock confirms that in policy design function the system did not change. The system was well-equipped before the crisis to handle a rapid income shock, at least in the short run.

Net redistribution increased during the crisis and was mainly driven by an increase in the generosity of benefits and a decrease in the concentration of benefits across the distribution (more people accessed them). These changes are mainly explained by the labour market shock, which signal the automatic stabilizers embedded in the existing system. The small effects due to changes in policy design confirm our previous finding that differences in policy design pre-during crisis are small.

### 5.4 International context

Where does Luxembourg stand among other countries in terms of its policy responses to the COVID-19 crisis and their ability to mitigate changes in individual welfare induced by the economic shock? In-depth studies focusing on the impact of the COVID-19 pandemic on the distribution of household incomes are relatively scarce and cover only a limited number of countries. Almeida et al. (2021) conducted a comparative study at the EU level by applying a reweighting methodology to calibrate based on economic forecasts for 2020 on GDP and employment and found substantial differences in the effect of COVID-19 and discretionary policy measures on equivalized disposable incomes, at risk of poverty rates and income inequality across EU countries. Our results on the stability of the income inequality and the resilience of the Luxembourg tax-benefit system in cushioning the shock resonate with their findings. The benefits received by workers in Luxembourg are relatively generous in an international comparison, offsetting a large part of the income shock (Almeida et al. 2021).

In what follows we reflect upon the role of tax-benefit systems and discretionary policy measures in cushioning income shocks in Luxembourg in comparison with the recent findings in Australia (Li et al. 2020), Ireland (O'Donoghue et al. 2020), Italy (Figari and Fiorio 2020), the UK (Brewer and Gardiner 2020; Brewer and Tasseva 2021), and Germany (Bruckmeier et al. 2021).

These countries differ in their sectorial composition of the economy, welfare systems and specific policy measures introduced in response to the COVID-19 crisis. With respect to the sectorial composition of the economy (Fig. 6), Italy relies heavily on the wholesale and retail trade, transport, and hospitality sectors, which are followed by manufacturing. Ireland has a substantial share of GDP produced in manufacturing, with the information and communication sector in the second place. The UK has the largest share of GDP produced in the public sector, which is followed by wholesale, retail, trade, transport and the hospitality services. In Germany, the largest share of gross value added is produced in the manufacturing sector, followed by the public sector and the wholesale and retail trade, transport, and hospitality sectors. The Luxembourgish economy, in contrast, is dominated by the service sector with the largest share of the gross value added coming from the financial and insurance services, followed by the public sector. Given the asymmetry of the COVID-19 crisis, which affects industries differentially, the consequences of this crisis for individual employment and market incomes vary substantially across countries.

From a welfare perspective, all five countries have well-established tax-benefit systems, which, to a larger or smaller extent, are expected to cushion income losses during an economic shock. The design of these systems, however, differs substantially across countries. Australia, Ireland and the UK belong to the Anglo-Saxon welfare regime, which puts an emphasis on the provision of means-tested benefit schemes aiming to reach those at the bottom of the income distribution (see Esping-Andersen 1990). In these countries, traditional unemployment benefit schemes are limited in duration, have a relatively low generosity (the replacement rates for a single person without children at 67% of the average wage fall between 50% and 60% for Ireland and the UK, and 40% for Australia - see Fig. 7), and

**Table 3** Decomposition of the change in Gini coefficients and in redistribution measures before (Q1) and during the COVID-19 crisis (April)

| | Gini Disposable (1) | Gini Gross Income (2) | Net Redistr. (3) | Benefit Regressivity (4) | Avg. Benefit Rate (5) | Tax Progressivity (6) | Avg. Tax rate (7) |
|---|---|---|---|---|---|---|---|
| 2020 Q1 | 0.260 | 0.484 | 0.224 | 0.910 | 0.354 | 0.315 | 0.165 |
| Changes under COVID-19 | | | | | | | |
| April - Q1 | 0.000 | 0.087 | 0.086 | −0.013 | 0.237 | 0.008 | −0.005 |
| Contribution of the shock and policy response | | | | | | | |
| LMS | 0.001 | 0.087 | 0.086 | −0.011 | 0.234 | 0.007 | −0.004 |
| TB | −0.001 | 0.000 | 0.001 | −0.002 | 0.003 | 0.000 | −0.000 |

Notes: LMS: Effect of the labour market shock assuming no tax-benefit changes pre-post Covid; TB: Effect of the difference in tax-benefit rules pre-post Covid in cushioning the shock

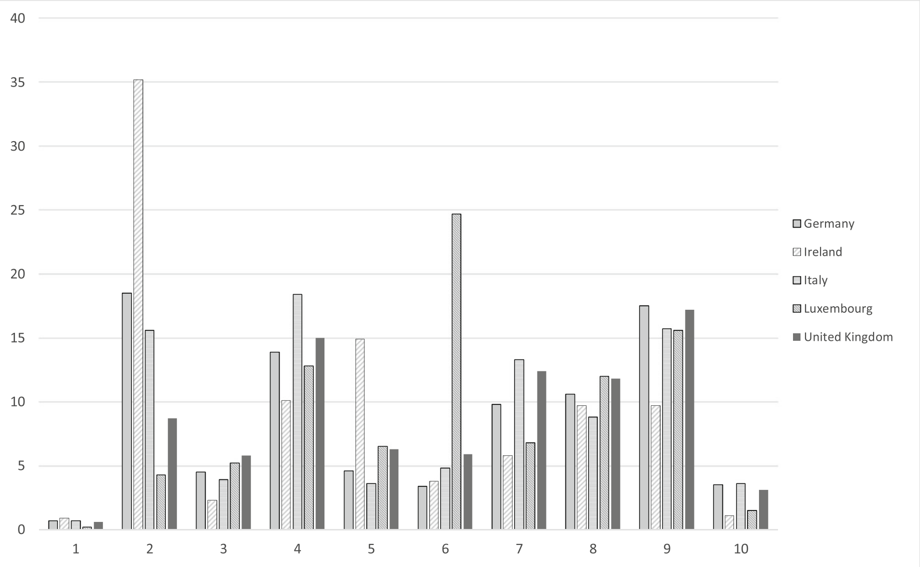

**Fig. 6** Gross value added by industry, selected European countries Note: 1 - agriculture, forestry and fishing; 2 - manufacturing; 3 - construction; 4 - wholesale and retail trade, transport, accommodation, and food services; 5 - information and communication; 6 - financial and insurance activities; 7 - real estate activities; 8 - professional, scientific, and technical activities; administrative and support service activities; 9 - public administration, defence, education, human health and social work activities; 10 - arts, entertainment and recreation, other service activities Source: Eurostat (extracted on July 17th 2021)

often rely on means-testing. Italy belongs to the Mediterranean welfare regime, where the provision of welfare is put mainly on families with a quite limited involvement of the state (for more details, see Ferrera (1996)). The main focus of the public welfare provision in Italy falls on pensions but it also supports incomes of individuals in need. As shown in Figure 7, the net benefit replacement rates are rather high in Italy, ranking it above the countries with Anglo-Saxon welfare systems. Germany, in contrast, belongs to the Continental welfare regime, which relies heavily on the provision of insurance-based social benefits with replacement rates at 60 percent. Luxembourg's welfare state is also classified under the Continental welfare regime grouping, but posts much higher replacement rates. The unemployment scheme in Luxembourg has a replacement of 80 to 85% of previous income during the first 12 months following the job loss (or higher when other benefits, such as housing, are considered). The country also provides relatively generous social assistance to individuals who exhausted the duration of their unemployment benefit payments, have limited incomes from other sources and can prove that they live in need.

As early research shows, the capacity of these welfare systems to cushion against the consequences of COVID-19 largely depends on their design. For example, Brewer and Tasseva (2021) show that the ability of UK automatic stabilizers to protect against income losses during a crisis is limited, when a substantial portion of employees loses jobs or is forced to reduce the number of working hours. The level of protection offered by Anglo-Saxon welfare states is particularly limited for middle and high earners. A similar finding is reported by Figari and Fiorio (2020), who conclude that the in-built features of the tax-benefit system in Italy were not sufficient to mitigate the economic shock induced by the COVID-19 crisis.

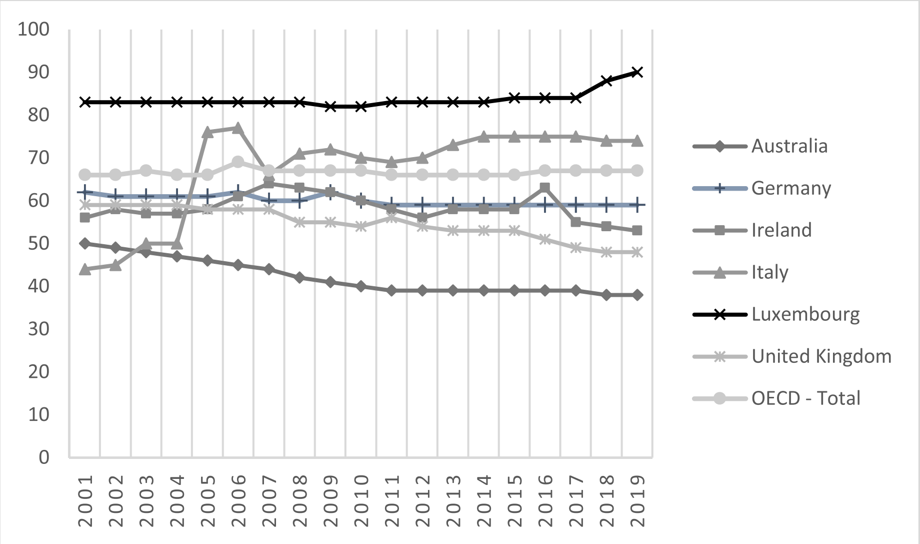

**Fig. 7** Net replacement rates in unemployment for a single person without children at 67% the average wage
Note: Figure based on OECD data, https://stats.oecd.org. The replacement rates include also social assistance and housing benefits

In Germany, Bruckmeier et al. (2021) found that the tax-benefit system acted as an important stabilizer during the COVID-19 crisis reducing losses in disposable household income and restraining an increase in inequality generated by the loss of labour market incomes. This impact was largely driven by the short-time work scheme.[29] As our results show, even without COVID-19 related policy responses, the Luxembourg tax-benefit system would have buffered the income losses of the affected individuals and households. Given that the system does not only target those at the bottom of the distribution, but supports individual incomes proportionally throughout the distribution, the activation of the system prevented an increase in disposable income inequality despite the surge in market income inequality.

Finally, all countries introduced discretionary policy measures in response to the COVID-19 crisis, for example, short-time work schemes or analogous schemes aimed to protect employment contracts in order to allow individuals to remain employed or re-start work as soon as the situation improves. Albeit they somewhat differ in terms of eligibility criteria, the replacement rates within these schemes are relatively high: 80% of the remuneration costs in Italy, Luxembourg, and the UK (subject to a maximum cap in all three countries), 60% of the last net salary (67% if there are children in the household) in Germany, a maximum rate of 410 per qualifying employee per week in Ireland (at least at the beginning of the crisis), a flat rate of 460 per week (A$750) in Australia. As shown by Brewer and Tasseva (2021), Figari and Fiorio (2020), Li et al. (2020), O'Donoghue et al. (2020), and Bruckmeier et al. (2021), short-time working schemes played a crucial role in mitigating market income losses in the UK, Italy, Australia, Ireland and Germany at the onset of the COVID-19 crisis. Australia has also temporarily doubled the unemployment benefit amount

[29]The authors, unfortunately, do not separate the impact of standard unemployment benefits scheme from the impact of short-time working scheme in the presentation of their results but stress that the buffering effect of tax-benefit system is mainly driven by the short-time work scheme.

and broadened the eligibility criteria, leading to a substantial increase in welfare expenditure. In countries such as Australia and Ireland, these discretionary measures led to a slight decrease in inequality of disposable household incomes. In Germany, apart from the short-time working scheme, the government introduced a set of discretionary non-employment measures (i.e. child bonus, support for single families, emergency child benefit supplement, etc.). As shown by Bruckmeier et al. (2021), these measures had a strong redistributive effect benefiting disproportionally those at the bottom of the distribution. While most of these countries introduced radically different policies from their existing systems, the COVID policies introduced in Luxembourg were minor modifications of existing policy instruments aimed to cushion the drops in individual incomes related to employment changes during the crisis. As our results indicate, in the absence of the COVID-induced policy response, the labour market shocks impact on household disposable incomes would have been absorbed by the automatic stabilisers (i.e. the already existing system of taxes and benefits).

These results, however, should be interpreted with caution. As the crisis evolves, those who were forced into short-time work might become unemployed and move to the classical unemployment scheme. This scheme will cushion their incomes with approximately the same replacement rate for another year but, then, those who fail to find new employment will exhaust the duration of unemployment benefits and will face the option of applying for social assistance, which is usually means-tested and involves stricter conditions. Additionally, it is also possible that the job market may experience major shifts as new work practices, such as remote working, may render some pre-COVID positions obsolete, creating frictions in the labour market. While the increased welfare expenditure has been effective in stabilising the income distribution in many countries, it is not clear whether some of the generous measures are sustainable given that the economy may take a few years to fully recover. The fiscal pressure along with the suppressed economic activities may force the government to either increase tax or reduce the generosity of the payments in the future, which may lead to a delayed increase in income inequality.

## 6 Conclusion

COVID-19 has swept across the globe in 2020, disrupting social and economic activities in most countries. In the early months of the crisis, full or partial lockdowns were put in place leading to job losses, which in turn impacted household incomes to varying degrees. The rapid economic changes and the lack of up-to-date data increased the importance of microsimulation tools for understanding the impact of the crisis and how the COVID-induced policy responses fared in cushioning household incomes. Our paper proposes a novel approach that combines microsimulation and nowcasting techniques to provide "near real-time" information to policy makers, to inform them about the impact that economic shocks and associated policy responses have on the distribution of household incomes. The approach proposed in this paper partially overcomes the unavoidable lags affecting the collection of survey data.

We contribute to the literature by applying this approach to produce evidence on the resilience of a typical Continental welfare regime during the COVID crisis. Luxembourg introduced minor tweaks to an existing tax-benefit system with a strong social insurance focus. Other countries, with an anti-poverty focus like the Anglo-Saxon welfare regime (e.g. Australia, the UK and Ireland) or with a social insurance regime focused on pensions such as the Mediterranean welfare regime (e.g. Italy), introduced radically different policies since the existing systems were not able to cope with the COVID-19 crisis.

Our analysis shows that, in Luxembourg, the crisis-induced policy reforms were effective in cushioning the impact of the crisis on household income across the distribution and in mitigating an increase in income inequality. We find evidence that disposable incomes dropped in absolute values throughout the distribution, more at the top than at the bottom. In relative terms, we find evidence of a U-shape relationship, with higher relative losses for the middle of the distribution compared to the top and bottom of the distribution. Relative to mean incomes, which dropped during the crisis, the poorest households improved their relative position compared to the households in the middle of the distribution, which lost relative to the mean. This resulted in a stable level of disposable income inequality across the different periods of 2020, albeit the composition of income sources changed: we observed a decrease in the share of labour market incomes compensated by an increase in the share of benefits, reflecting the cushioning effect of the transfer system.

Under the impact of the labour market shock, labour incomes (and overall market incomes) dropped throughout the distribution and became more unequally distributed (by 5.3 Gini points in Q2 and 8.7 Gini points in April). The disequalizing evolution in labour incomes was overpowered by an increase in redistribution through the tax-benefit system.

We isolate the impact of the COVID-induced policy reforms from the impact of the labour market shock to see how the pre-COVID tax-benefit system would have fared against the shock in comparison to the reformed tax-benefit system. We find evidence that the system was well-equipped ahead of the crisis to cushion household incomes against job losses. We find inconsequential distributional differences between the two systems (before and during the crisis) when assessing the functioning of both systems during the same labour market shock. The main difference between the pre-existing unemployment benefit and the COVID-19 short-time working scheme lies in the speed and eligibility under which individuals could access benefits: people falling into short-time work could access benefits faster than under the standard unemployment scheme.[30]

Net redistribution increased during the crisis. This was mainly driven by the generosity of benefits and improved access to benefits. Increases in net redistribution are mainly explained by the large labour market shock, reflecting the automatic stabilizers embedded in the pre-COVID system.

On a methodological note, our approach could serve as a near real-time analysis and decision support tool to monitor the recovery, with high applicability for policy makers. It is applicable to assessing the impact of later stages in the COVID crisis such as re-opening strategies and their long-term sustainability. The methodology is adequate to explore these

[30]This paper focuses on the day-after effects of COVID-19 on the distribution of household disposable income. Short-time work (STW), the primary instrument supporting workers in Luxembourg throughout the pandemic, however has important labour market effects beyond replacing earnings. One of the main objectives of the STW scheme is to help employers meet their liquidity needs without resorting to layoffs. By maintaining the employment link, STW supports employers in preserving their human capital and minimizes recruitment costs in the future. Workers remain legally employed and can restart working as soon as the economic situation improves without searching for a new job. In the absence of STW, workers that lost their job benefit from the unemployment benefit or the social assistance benefit. Unemployment and social assistance benefits only provide income replacement and do not support firms in meeting their liquidity needs or maintain employment relations. The conclusions of this paper should therefore be interpreted with caution. Existing automatic stabilizers were adequate in protecting households from immediate income losses. We do however not draw any conclusions on the impact of the COVID-19 policy response, notably STW, on the labour market beyond day-after effects.

changes both for the whole economy and for specific sectors. Using the model, policymakers could explore a menu of policy combinations in terms of their impact on key welfare measures and fiscal costs. Furthermore, this approach could be linked to epidemiological models (e.g. Burzynski et al. 2020) which allow extending the scope of the analysis to adjacent dimensions, such as the roll-out of vaccines and recovery strategies. As the infrastructure relies on a flexible income generation model, on comparative cross-national data sources, and on the pan-European tax-benefit model, EUROMOD, it is scalable to other countries thereby enhancing its applicability and policy relevance.

**Supplementary Information** The online version contains supplementary material available at doi:10.1007/s10888-021-09524-4.

**Acknowledgements** This research is part of the project *Modelling the macroeconomic and distributional effects of COVID-19 and restarting scenarios* supported by the National Research Fund, Luxembourg (grant **COVID-19/2020-2/14854549/MODVid**). We would like to acknowledge the data support of Johann Neumayr, Marco Schockmel, Mehran Kafaï, Paul Reiff, Véronique Sinner and Jérôme Hury at Statec, and Joel Machado and Kristell Leduc at LISER. The results use EUROMOD version I2.0+, which is maintained, developed and managed by the Joint Research Centre (JRC) of the European Commission, in collaboration with EUROSTAT and national teams from the EU countries. The results and their interpretation are our responsibility. We are grateful for comments from Frédéric Docquier, Karina Doorley, from participants of the Research Colloquium at Trier University, the IMA Conference on "Microsimulation modelling of policy responses to COVID-19", the joint LISER-NUIG-IMA Microsimulation and Inequality Seminar and the ISER Online Expert meeting on nowcasting and mid-term projections through microsimulation models. The data that support the findings of this study are available from EUROSTAT, but restrictions apply to the availability of these data, which were used under license for the current study, and so are not publicly available. Data are however available from the authors upon reasonable request and with permission of EUROSTAT.

**Competing Interests Statement** The authors of this research certify that they have no conflict of interests in the work discussed in this manuscript.

**Open Access** This article is licensed under a Creative Commons Attribution 4.0 International License, which permits use, sharing, adaptation, distribution and reproduction in any medium or format, as long as you give appropriate credit to the original author(s) and the source, provide a link to the Creative Commons licence, and indicate if changes were made. The images or other third party material in this article are included in the article's Creative Commons licence, unless indicated otherwise in a credit line to the material. If material is not included in the article's Creative Commons licence and your intended use is not permitted by statutory regulation or exceeds the permitted use, you will need to obtain permission directly from the copyright holder. To view a copy of this licence, visit http://creativecommons.org/licenses/by/4.0/.

## Electronic supplementary material

Below is the link to the electronic supplementary material.

(PDF 1.37 MB)

## Data Availability

The analysis conducted in this study is based on EU-SILC data standardized to be used with the EUROMOMD tax-benefit microsimulation model. The data cannot be shared. Access to the data can be obtained by submitting a request to EUROSTAT.
