# Supplementary material for: The COVID-19 resilience of a continental welfare regime - nowcasting the distributional impact of the crisis
Source: J Econ Inequal. 2022 Feb 22;20(4):777–809. doi: 10.1007/s10888-021-09524-4 (PMC8861260; doi:10.1007/s10888-021-09524-4)
Supplement: Supplementary file 1 — (PDF 1.37 MB) [file 10888_2021_9524_MOESM1_ESM.pdf]

Appendix

A Discretionary Policies and Automatic stabilizers

Table A–1: Percentage of employees benefiting from the short-time working scheme among the total number of employees residing in Luxembourg, by industry

Industry	Percentage of workers (hours) on short-time work			
	April 2020	Q2 2020	Q3 2020	Q4 2020
Agriculture, forestry, fishery	23.8	10.1	0.0	0.0
	(12.4)	(5.4)	(0.2)	(0.5)
Manufacturing, mining, quarrying and turf production, electricity, gas and water supply	46.9	28.6	17.9	23.0
	(19.7)	(13.0)	(3.2)	(3.0)
Construction	87.0	33.3	1.1	1.4
	(49.9)	(18.4)	(0.3)	(0.5)
Wholesale and retail	52.8	29.2	4.7	5.8
	(33.3)	(18.4)	(1.8)	(2.3)
Hotels and restaurants	87.9	71.2	31.1	40.8
	(69.3)	(54.6)	(15.1)	(26.9)
Transport and communication	25.5	16.8	7.8	6.8
	(9.3)	(7.7)	(2.1)	(1.5)
Financial intermediation	0.4	0.2	0.0	0.0
	(0.9)	(0.6)	(0.1)	(0.1)
Real estate and business	31.8	17.6	4.6	4.2
	(16.3)	(10.2)	(1.9)	(1.6)
Public administration and defence	0.0	0.0	0.0	0.0
	(0.1)	(0.0)	(0.0)	(0.0)
Education	3.0	1.9	0.4	0.6
	(10.2)	(5.9)	(0.8)	(0.9)
Health and social work	5.7	3.1	0.5	0.4
	(3.4)	(1.7)	(0.1)	(0.2)
Other	26.9	18.5	5.5	8.3
	(28.7)	(17.7)	(2.9)	(5.7)
Total	33.7	18.6	5.5	6.4
	(20.1)	(11.4)	(1.9)	(2.4)

Note: Data provided by Statec. 'Other' contains NACE Rév.2 categories (share of workers on short-time work for April, Q2, Q3 and Q4 respectively in brackets): R - Arts, entertainment and recreation (27.7%; 20.9%; 8.2%; 15.7%), S - Other service activities (49.5%; 32.6%; 8.5%; 15.7%), T - Activities of households as employers; undifferentiated goods- and services- producing activities of households for own use (1.0%; 0.5%; 0.0%; 0.0%)

Table A–2: Policy instruments activated to stabilize the economic situation and cushion individual incomes

Economic Stabilization Program - March 18th		
Policy instrument	Description	Beneficiaries
Short-time work scheme (chômage partiel en cas de force majeure)	The state takes over 80% of the remuneration of employees for hours not worked, if the employee had their working hours temporary reduced, up to a maximum of 2.5x the social minimum wage for unskilled workers (€5354,95)	Startups/SME/large enterprises/non-for-profit organizations
Special family leave	The state takes over 100% of the remuneration of employees that interrupt their work to care for children under 13 due to the closure of educational establishments, or due to quarantine.	Employees/self-employed
Special sick leave	National Health Fund (CNS) takes over 100% of the remuneration costs for individuals sick with COVID-19 from the first day of their illness	SME/self-employed, large enterprises
Néistart Lëtzebuerg/ Economic Recovery Plan - June until December 1st		
Progressive transition from short-time work "en cas de force majeure" to structural short-time work	Continued support to businesses previously benefiting from short-time work "en cas de force majeure". Eligibility is differentiated based on industry with simplified procedure for businesses operating in vulnerable sectors.	Businesses that benefitted from short-time work "en cas de force majeure"
Expensive life allowance (allocation de vie chère)	Doubling of the expensive life allowance, from a monthly amount of 110 Euros to a monthly amount of 220 Euros	Low-income households
Main existing automatic stabilizers		
Unemployment payment (indemnité de chômage)	Registered job seekers benefit from a 80% compensation of their gross salary (average of last 3 months of employment), if they made sufficient social security contributions. The payment is increased to 85% if the person has one or more children.	Unemployed residents
REVIS (Social Inclusion Income)	Means-tested flat rate monthly social assistance payment of €1501,65 for the first adult, €750,95 for any additional adults. Families with children receive a top up of €112,78 per month and an additional payment of €233,32 per child per month. Single parents receive a top-up of €68,96 per month. Individuals participating in activation measures can access additional "activation payments".	Residents older than 24 years
Expensive life allowance (allocation de vie chère)	Means-tested flat-rate payment of €110 monthly supporting families facing high living costs	Resident Low-income households

Source: Compilation of rules from www.guichet.lu

Note: *As part of the policy response to COVID-19, the Luxembourg Government simplified access to certain benefits for individuals and firms. Individuals benefitted from simplied access to unemployment payments, suspended social insurance contribution requirements, the requirement to submit physical copies of administrative documents and to visit the unemployment office in person. Firms benefitted from simplified access to short-time work; reducing information requirements (i.e. the provision of a recovery plan). Waiting periods for the reimbursement of Illness benefits and Family leave were suspended.*

B Method

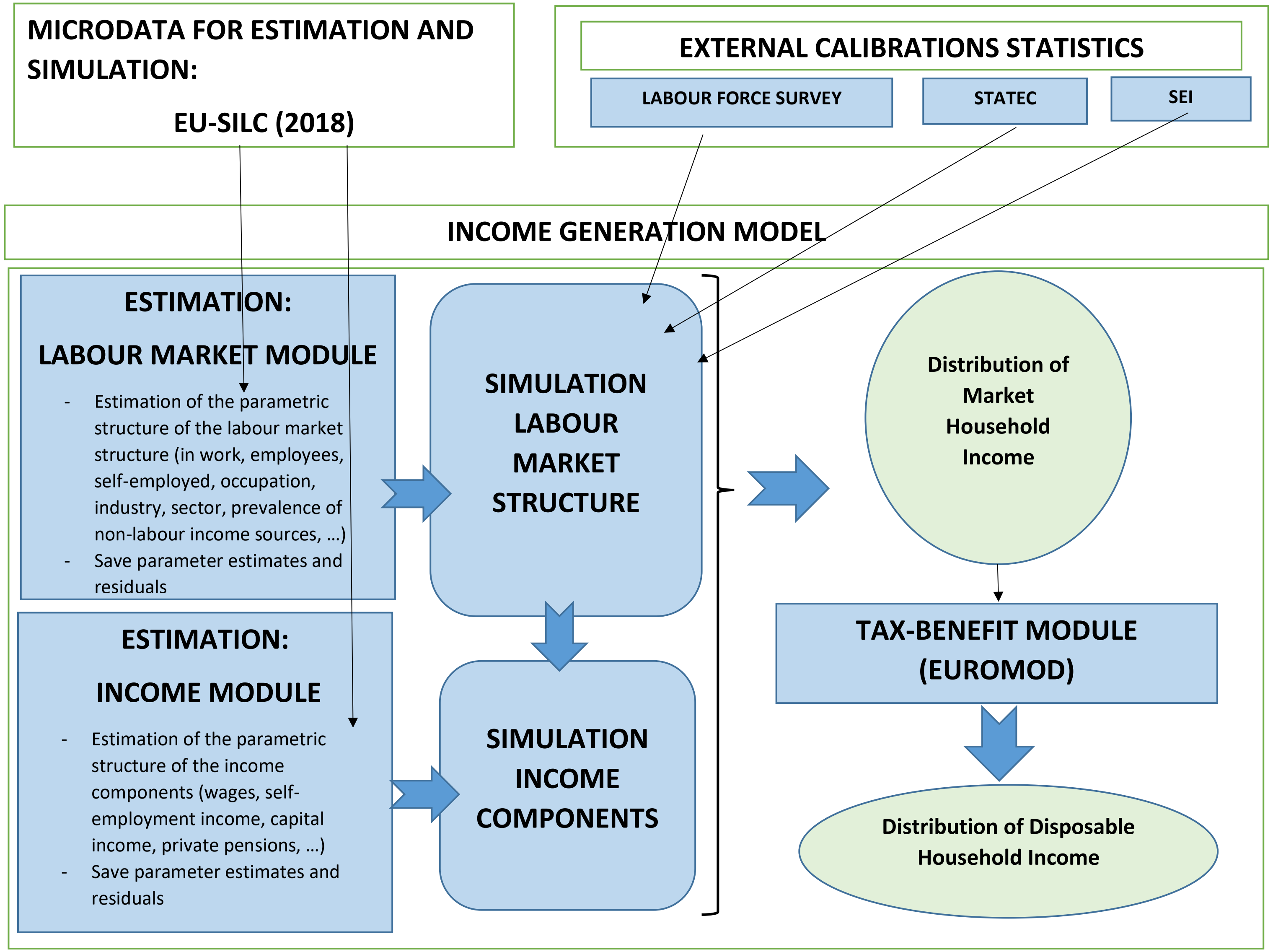

Figure B–1: Summary chart of the nowcasting framework

Note: European Union Statistics on Income and Living Conditions (EU-SILC), National Statistical Office (STATEC), Survey on the Socio-Economic Impact of the COVID-19 Crisis (SEI)

C Models

Table C–3: Definition of income components and summary modelling information - part 1

Variable	Definition	Level	Treatment	Transformation	Model
y_h	total household disposable income	household	aggregate		–
y_h^L	gross labour income	household	aggregate		–
I_{hi}^{emp}, y_{hi}^{emp}	employee income (wage*hours)	individual	modelled	LM struct/Returns	logit, Singh-Maddala
I_{hi}^{se}, y_{hi}^{se}	self-employment income (receipt, amount)	individual	modelled	LM struct/Returns	logit, log-linear
y_h^K	capital income (investment, property)	household	aggregate		–
I_h^{inv}, y_h^{inv}	investment income (receipt, amount)	individual	modelled	LM struct/Returns	logit,log-linear
I_h^{prop}, y_h^{prop}	property income (receipt, amount)	individual	modelled	LM struct/Returns	logit,log-linear
I_h^{pripen}, y_h^{pripen}	private pensions (receipt, amount)	individual	modelled	LM struct/Returns	logit,log-linear
y_h^O	other non-benefit incomes (receipt, amount)	individual	aggregate, modelled	LM struct/Returns	logit, log-linear

Table C–4: Definition of income components and summary modelling information - part 2

Variable	Definition	Level	Treatment	Transformation	Model
y_h^B	public transfers	household	aggregate	TB	–
y_{hi}^{repl}	replacement income (pensions, unemployment)	individual	aggregate	TB	–
I_{hi}^{unemp}, y_{hi}^{unemp}	unemployment benefits (receipt, amount)	individual	aggregate, modelled	TB	logit,log-linear, EUROMOD
$I_{hi}^{partialunemp}$, $y_{hi}^{partialunemp}$	short-time work - Covid benefit (receipt, amount)	individual	modelled	LM/TB	logit, EUROMOD
I_{hi}^{pens}, y_{hi}^{pens}	public (state, survival, occupational pensions) (receipt, amount)	individual	aggregate, modelled	TB	logit,log-linear, EUROMOD
$I_{hi}^{disability}$, $y_{hi}^{disability}$	disability (receipt and amount) sickness (receipt and amount)	individual	aggregate, modelled	TB	logit, log-linear, EUROMOD
$I_{hi}^{sickness}$, $y_{hi}^{sickness}$	sickness (receipt, amount)	individual	modelled	TB	logit, log-linear, EUROMOD
$I_h^{housing}$, $y_h^{housing}$	housing benefits (receipt, amount)	household	modelled	TB	logit, log-linear, EUROMOD
y_h^{sa}	social assistance	household	modelled	TB	EUROMOD
y_h^{osw}	other social welfare	household	modelled	TB	logit, log-linear, EUROMOD
y_h^{fb}	family benefits	household	modelled	TB	EUROMOD
y_h^{mb}	maternity benefit	household	modelled	TB	logit,log-linear, EUROMOD
y_h^{cb}	child benefit	household	modelled	TB	EUROMOD
t_h	taxes and social security contributions	household	aggregate, modelled	TB	EUROMOD

Table C–5: Demographic and labour market variables

Variable	Definition	Level	Treatment	Factor	Model
n_h	household size	household	observed	Demo	–
x_h	household-level demographic characteristics (number of children aged 0–3, 4–11 and 12–15) and individual characteristics of the household head (marital status, gender, age and age squared, university education)	household	observed	Demo	–
x_{hi}	individual-level characteristics: gender, age and age squared, university education, marital status, number of children in the household (aged 0–3, 4–11 and 12–15), citizenship, age*university, age squared*university	individual	observed	Demo	–
occ_{hi}	Occupation (1-digit ISCO); for working individuals only	individual	modelled	LM Struct	multinomial logit
ind_{hi}	Industry (8 categories); for working individuals only	individual	modelled	LM Struct	multinomial logit
pub_{hi}	Public or private sector job; for employees only	individual	modelled	LM Struct	logit
$white_{hi}$	White collar vs. blue collar	individual	modelled	LM Struct	logit
$experience_{hi}$	Labour market experience	individual	observed	LM Struct	–
s_{hi}	Number of hours worked	individual	modelled	LM Struct	linear
w_{hi}	Average wage rate; for employees only	individual	modelled	Returns	Singh-Maddala

D Population and labour market structure

Table D–6: Population and labour market structures (shares of total population)

	Q1 2020	April 2020	Q2 2020	Q3 2020	Q4 2020
Demographic					
Tertiary Education	0.338	0.338	0.338	0.338	0.338
People 16-65	0.704	0.704	0.704	0.704	0.704
People >65	0.133	0.133	0.133	0.133	0.133
Child 0-3	0.042	0.042	0.042	0.042	0.042
Child 4-11	0.077	0.077	0.077	0.077	0.077
Child 12-15	0.044	0.044	0.044	0.044	0.044
Married	0.545	0.545	0.545	0.545	0.545
Citizen	0.541	0.541	0.541	0.541	0.541
Male	0.498	0.498	0.498	0.498	0.498
Labour market					
In-work	0.579	0.573	0.574	0.573	0.585
Employee/Self-Employed	0.908	0.898	0.897	0.900	0.908
Covid Partial Unemployment	.	0.285	0.173	0.047	0.053
Occupation					
Managers	0.059	0.070	0.069	0.059	0.056
Professionals	0.413	0.428	0.428	0.420	0.434
Associate Prof.	0.162	0.157	0.157	0.171	0.172
Clerks	0.074	0.074	0.074	0.072	0.068
Service	0.098	0.093	0.093	0.095	0.095
Craft	0.086	0.094	0.094	0.089	0.086
Plant	0.063	0.043	0.043	0.050	0.057
Unskilled	0.045	0.042	0.043	0.044	0.030
Industry					
Agriculture	0.016	0.016	0.016	0.017	0.016
Manufacturing...	0.046	0.049	0.049	0.052	0.044
Construction	0.079	0.084	0.084	0.075	0.083
Commerce	0.396	0.404	0.404	0.410	0.419
Transport	0.057	0.051	0.052	0.050	0.057
Public administration	0.114	0.122	0.122	0.119	0.113
Education/Health/Social	0.214	0.210	0.210	0.223	0.215
Other	0.079	0.063	0.063	0.053	0.053
Public/Private	0.061	0.059	0.059	0.060	0.060
Other market factors					
With private pensions	0.005	0.005	0.005	0.005	0.005
With capital income	0.350	0.350	0.350	0.350	0.350
With other income	0.013	0.013	0.013	0.013	0.013

Notes: The estimates are weighted. The shares for education refer to age-group 25-64; for married, sex to age >= 16; for in-work to ages 15 to 80; for employees, occupation, industry and sector to those in work aged [16, 80); for citizen to the entire sample. The shares for private pensions refer to ages >= 45, for capital age>= 16. The aligned shares for inwork, employees, occupation, industry and sector include people in partial unemployment due to COVID as the LFS considers their status as employed. In order to capture the labour market shock, the shares in temporary unemployment reflect the proportion of those employed aged [16,80) who fell into partial unemployment. Wholesale and retail, Hotels and restaurants, Communication, Financial intermediation, and Real estate and business were grouped into âCommerce â. âOtherâ contains the following NACE categories: R - Arts, entertainment and recreation, S - Other service activities, T - Activities of households as employers; undifferentiated goods- and services-producing activities of households for own use, U - Activities of extraterritorial organizations and bodies, and NRP - No response.

E Inequality decomposition by income source

In this Appendix we evaluate the contribution of various income sources to the Gini coefficient and its change over time following the decomposition procedure of Lerman and Yitzhaki (1985). This procedure foresees partitioning of the Gini coefficient in a set of components associated with contributions of various income sources, where the contribution of each income source is defined as a product of its share in total income (s), inequality measured with the Gini coefficient (g), and correlation with total income (r). Among income sources, we consider labour income, private pensions, capital and other market incomes, taxes, social security contributions, and public benefits (where we separate public pensions from the remaining public transfers, such as unemployment benefits, sickness benefits, family benefits, housing benefits, social assistance). The results of this decomposition exercise are presented in Table E–7.

We find that labour income, pensions, capital and other incomes contribute positively to the level of income inequality in Luxembourg, whereas, benefits, taxes, and social security contributions have an equalising effect, offsetting a substantial portion of the increase in the Gini associated with labour income, capital income, and both types of pensions. The composition of disposable income changed substantially over the crisis, whereas the level of inequality stayed roughly unchanged. At the beginning of the crisis (in April 2020 and overall over Q2) we observe a decrease in the share of labour incomes compensated by an increase in the share of benefits, reflecting the cushioning effect of the transfer system. Labour income became more unequally distributed (the figures include zeros which resulted from job losses) and more concentrated at the top of the distribution of disposable income, whereas benefits became less concentrated (meaning more people along the distribution access benefits during the crisis). The equalizing contribution of benefits more than doubled in April and increased by 79 percent in Q2 as compared to the pre-crisis level reflecting a partial substitution of labour incomes with unemployment benefits. In Q3 and Q4, the situation stabilized and the contributions of various income sources to inequality came back to the pre-crisis level.

Figure E–2 shows the changes in labour market and benefit incomes along the distribution of household disposable income. Looking at the evolution of labour market incomes first, one can see that they declined substantially in the first month of the crisis, with the decline being the smallest at the bottom and the top of the distribution and somewhat larger between the 20th and 60th percentiles. This reflects the trend in the distribution of disposable income depicted in Figure 4. As a compensation for the decline in market incomes, benefits increased in size along the entire distribution of household income. The size of the increase was proportional to the decline in labour incomes being the largest in the middle of the distribution. The trends look similar in April and in the second quarter of 2020 but the size of income changes are slightly larger at the peak of the crisis as compared to Q2 in general. In Q3 and Q4, the levels of labour and benefit incomes along the distribution returned to the pre-crisis level.

Figures E–3 and E–4 provide further evidence on the type of benefits prevailing in the benefits portfolio before the crisis and at the beginning of the crisis. Figure E–3 shows that, in terms of the size, both COVID-related and total benefits (without pensions) were the highest at the peak of the crisis (in April) and declined during Q2. In general, COVID benefits took the largest share in total benefits (without pensions) at the moment of the crisis outbreak but this share declined as the crisis evolved. When looking at the composition of benefits including pensions (Figure E–4), one can see that COVID benefits constituted around 25% of total benefits in April but their share dropped to around 17 percent already in Q2.

Table E–7: Household Equivalized Disposable Income Decomposition by Income Source (Euro) - Q1 vs. April, Q2, Q3, Q4

Country	Share (s)	Gini (g)	Correlation (r)	Concentration (c=g*r)	Relative Contribution (s*g*r/G)
Q1					
Labour Income	0.953	0.503	0.749	0.377	1.382
Private Pensions	0.000	0.998	0.293	0.293	0.000
Capital and other	0.031	0.942	0.613	0.578	0.069
Public Pensions	0.258	0.826	0.407	0.337	0.334
Benefits	0.103	0.652	-0.376	-0.245	-0.097
Taxes	-0.220	0.658	0.957	0.630	-0.534
SIC	-0.124	0.398	0.806	0.321	-0.154
Total		0.260			
April					
Labour Income	0.795	0.594	0.747	0.444	1.360
Private Pensions	0.000	0.998	0.344	0.343	0.000
Capital and other	0.032	0.942	0.628	0.592	0.074
Public Pensions	0.268	0.826	0.453	0.375	0.387
Benefits	0.240	0.623	-0.251	-0.156	-0.145
Taxes	-0.213	0.665	0.960	0.638	-0.522
SIC	-0.124	0.405	0.797	0.323	-0.153
Total		0.260			
Q2					
Labour Income	0.861	0.560	0.750	0.420	1.385
Private Pensions	0.000	0.998	0.324	0.323	0.000
Capital and other	0.032	0.942	0.615	0.580	0.071
Public Pensions	0.264	0.826	0.431	0.356	0.360
Benefits	0.184	0.650	-0.290	-0.189	-0.133
Taxes	-0.217	0.663	0.959	0.636	-0.529
SIC	-0.124	0.404	0.800	0.323	-0.154
Total		0.261			
Q3					
Labour Income	0.926	0.523	0.748	0.391	1.385
Private Pensions	0.000	0.998	0.303	0.302	0.000
Capital and other	0.031	0.942	0.616	0.580	0.070
Public Pensions	0.259	0.826	0.415	0.343	0.340
Benefits	0.128	0.663	-0.328	-0.217	-0.106
Taxes	-0.220	0.662	0.958	0.634	-0.535
SIC	-0.124	0.402	0.804	0.324	-0.154
Total		0.262			
Q4					
Labour Income	0.932	0.519	0.749	0.389	1.394
Private Pensions	0.000	0.998	0.289	0.289	0.000
Capital and other	0.031	0.942	0.606	0.571	0.068
Public Pensions	0.256	0.826	0.402	0.332	0.327
Benefits	0.127	0.668	-0.300	-0.200	-0.098
Taxes	-0.223	0.656	0.958	0.629	-0.538
SIC	-0.125	0.398	0.805	0.320	-0.153
Total		0.260			

Notes: Nowcasted distribution in Luxembourg for Q1, April and Q2(Euromod output) Labour Income = Employment + Self-Employment; Capital Income = Investment + Property; Other Income = other + private transfers.

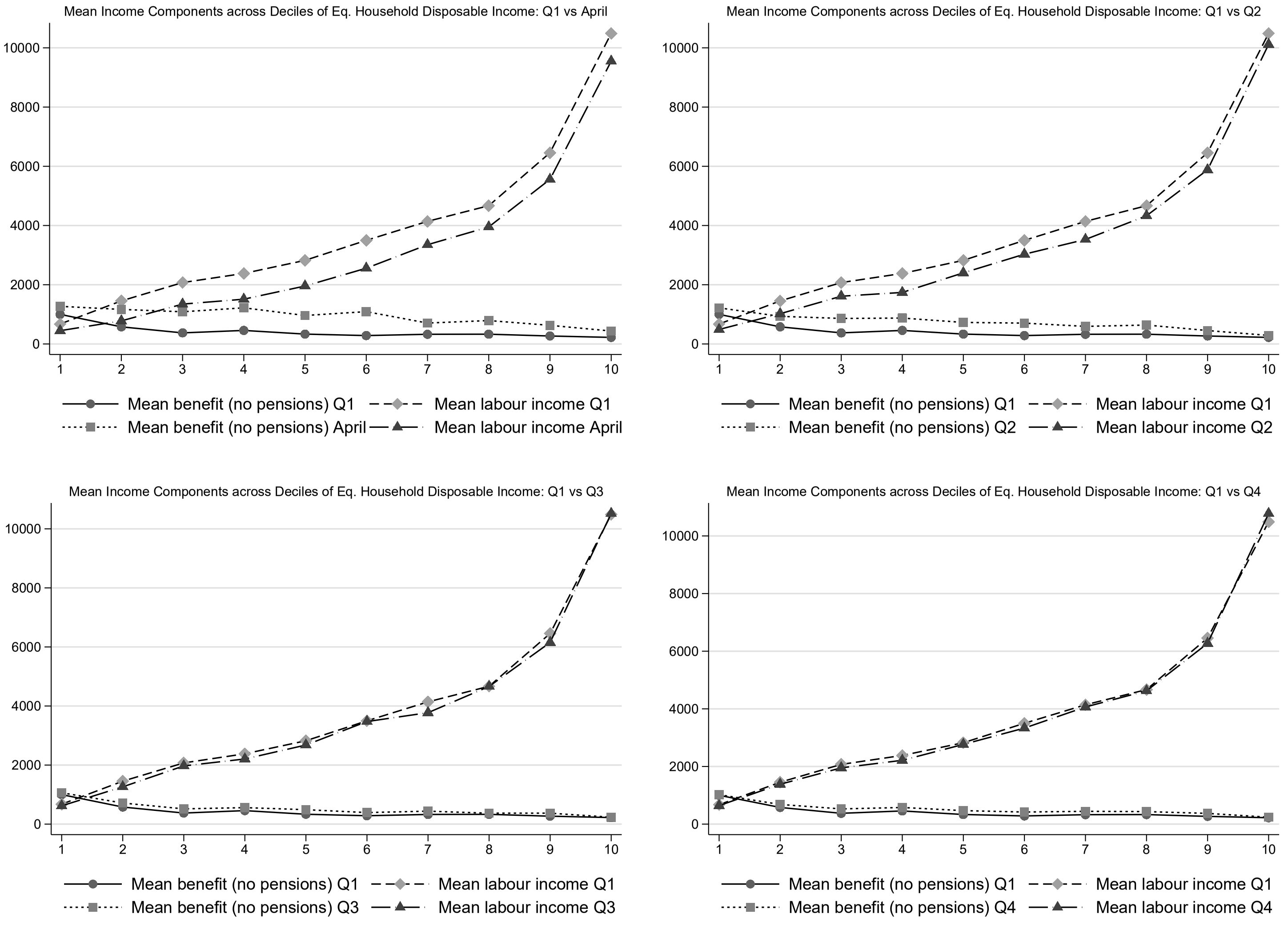

Figure E–2: Mean income components by deciles of equivalized household disposable income: Q1(pre-COVID) vs April, Q2, Q3, Q4 nowcasted
Note: Both income components are in gross values. The means refer to monthly averages.

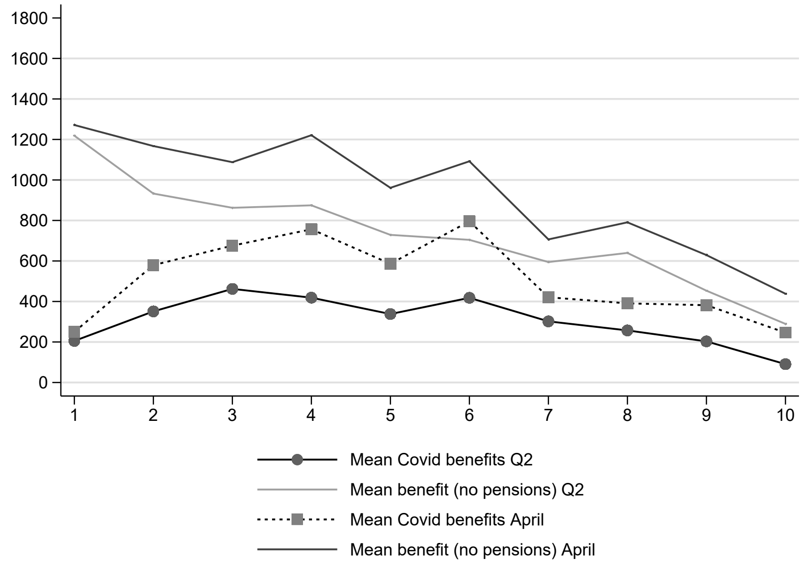

Figure E–3: Mean COVID equivalized benefits by deciles of equivalized household disposable income: Q1(pre-COVID) vs April, Q2, Q3, Q4 nowcasted
Note: Both components are in gross values. The means refer to monthly averages.

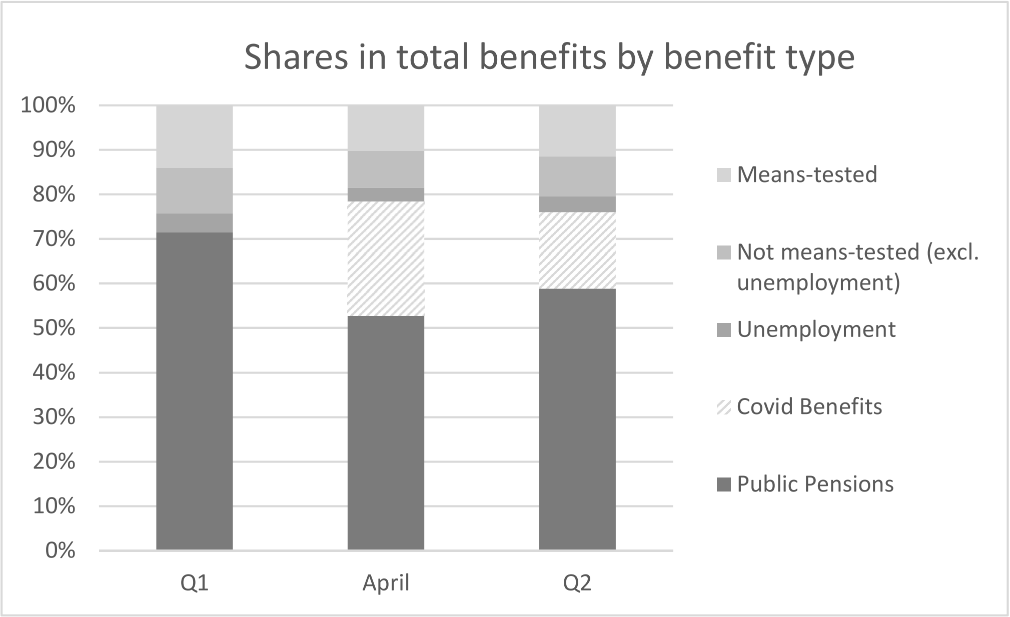

Figure E–4: Shares in total benefits by benefit type: Q1(pre-COVID) vs April, Q2 nowcasted
Note: The shares are based on gross equivalized values (monthly averages).

F Decomposition results

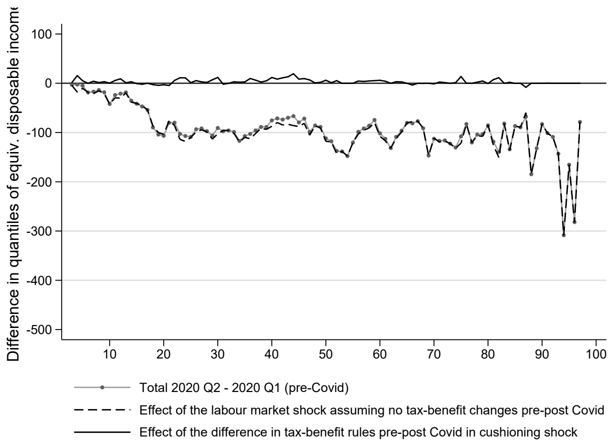

(a) Absolute decomposition: Q2

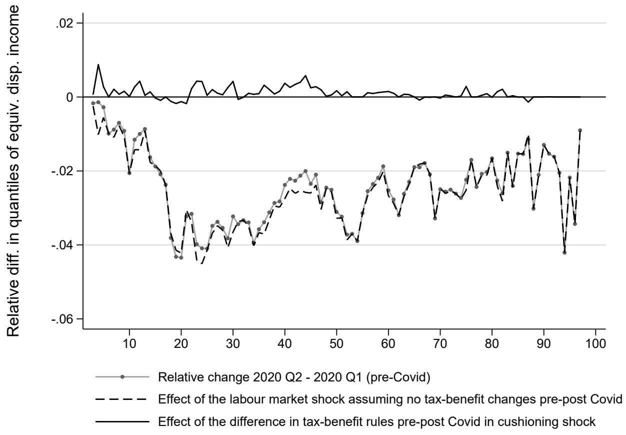

(b) Relative decomposition: Q2

Figure F–5: Contribution of the labour market shock and policy response to the change in income quantiles under the COVID crisis: Q2-Q1

Table F–8: Decomposition of the change in Gini coefficients and in redistribution measures before (Q1) and during the COVID-19 crisis (Q2)

	Gini Disposable (1)	Gini Gross Income (2)	Net Redistr. (3)	Benefit Regressivity (4)	Avg. Benefit Rate (5)	Tax Progressivity (6)	Avg. Tax rate (7)
2020 Q1	0.260	0.484	0.224	0.910	0.354	0.315	0.165
Changes under COVID-19							
Q2 - Q1	0.002	0.053	0.052	-0.003	0.128	0.004	-0.002
Contribution of the shock and policy response							
LMS	0.002	0.053	0.051	-0.003	0.127	0.004	-0.002
TB	-0.000	0.000	0.000	-0.001	0.001	0.000	-0.000

Notes: LMS: Effect of the labour market shock assuming no tax-benefit changes pre-post Covid; TB: Effect of the difference in tax-benefit rules pre-post Covid in cushioning the shock.
